# Phase transitions in *in vivo* or *in vitro* populations of spiking neurons belong to different universality classes

**Published:** 2025-05-13

**Authors:** Braden A. W. Brinkman

**Affiliations:** 1 Department of Neurobiology and Behavior, Stony Brook University, Stony Brook, NY, 11794, USA

## Abstract

The “critical brain hypothesis” posits that neural circuitry may be tuned close to a “critical point” or “phase transition”—a boundary between different operating regimes of the circuit. The renormalization group and theory of critical phenomena explain how systems tuned to a critical point display scale invariance due to fluctuations in activity spanning a wide range of time or spatial scales. In the brain this scale invariance has been hypothesized to have several computational benefits, including increased collective sensitivity to changes in input and robust propagation of information across a circuit. However, our theoretical understanding of critical phenomena in neural circuitry is limited because standard renormalization group methods apply to systems with either highly organized or completely random connections. Connections between neurons lie between these extremes, and may be either excitatory (positive) or inhibitory (negative), but not both. In this work we develop a renormalization group method that applies to models of spiking neural populations with some realistic biological constraints on connectivity, and derive a scaling theory for the statistics of neural activity when the population is tuned to a critical point. We show that the scaling theories differ for models of *in vitro* versus *in vivo* circuits—they belong to different “universality classes”—and that both may exhibit “anomalous” scaling at a critical balance of inhibition and excitation. We verify our theoretical results on simulations of neural activity data, and discuss how our scaling theory can be further extended and applied to real neural data.

There is little hope of understanding how each of the $𝒪\left(10^{11}\right)$ neurons contributes to the functions of the brain [1]. Even individual brain regions contain millions of neurons [2, 3], more than can be individually mapped out, but enough that the tools of statistical physics can be applied to understand how collective patterns of neural activity may contribute to brain function. Indeed, experimental work has demonstrated that neural circuitry can operate in many different regimes of collective activity [4–8]. Theoretical and computational analyses of these collective dynamics suggest that transitions between different operating regimes may be sharp, akin to phase transitions observed in statistical physics [4, 6, 9–17]. The theory of critical phenomena predicts that at a phase transition the statistical fluctuations of a system span many orders of magnitude in space and time, and the system displays approximate scale invariance [18]. In the brain, scale invariance would lead not only to power-law scaling in neural activity, but *scaling collapse*, in which activity data collected under different conditions can be systematically rescaled to fall onto a single universal curve. Such signatures of criticality have been observed in neural data from the retina [19], visual cortex [20, 21], hippocampus [22], and other cortical areas [23]. These observations have lead neuroscientists to hypothesize that circuitry in the brain is actively maintained close to critical points—the dividing lines between phases; this has become known as the “critical brain hypothesis” [8, 24–26]. Proponents of this hypothesis argue that these scale-spanning fluctuations could benefit brain function by minimizing circuit reaction times to perturbations, facilitating switches between computations and maximizing information transferred [25].

However, our understanding of critical phenomena and scaling in neural systems is largely phenomenological, based on analogies with well-studied systems from physics that lack many of the biological features of neural circuits, such as complex network structure and distinct excitatory and inhibitory cell types. This makes it difficult to resolve apparently inconsistent measurements of signatures of criticality across different brain areas. For example, some analyses of neural data appear to suggest that power law exponents are of the “mean-field” type, predictable by standard dimensional analysis, while other studies point towards anomalous exponents that that deviate from the mean-field predictions [8, 27, 28]. Anomalous power law exponents come in sets corresponding to different “universality classes,” where the universality class of a system is characterized by symmetries of the dynamics and statistical distributions, and, in lattices and continuous media, the dimension of the system. While universality classes and anomalous scaling are well understood theoretically in lattices and continuous media, the situation on more general networks remains an open problem.

The modern understanding of critical phenomena, and the origin of anomalous scaling, is based on the renormalization group (RG). The RG is a framework for organizing activity into a hierarchy of scales and determining how statistical fluctuations at each scale contribute to the overall statistics of a system. In soft condensed matter physics, these scales are typically distance and time, and the RG reveals how microscopic details influence dynamics and statistics on long spatial and temporal scales. However, it is not clear what the appropriate scales are in neural circuits, as spatial distance between neurons does not necessarily reflect the influence they have on each other through chains of synaptic connections.

Recent work has used ideas from the RG to devise phenomenological schemes for analyzing data, determining that a system is critical if the data can be shown to be approximately scale invariant under repeated coarse-graining of the principal components of neural covariances [22, 29–31] or in time [32]. However, a theoretical understanding of neural systems through the lens of the RG has so far been restricted to models from statistical physics that are re-interpreted in terms of of coarse-grained neural signals, such as active/inactive units [16, 29], or in networks of neurons described by firing rates [33, 34], rather than populations of neurons that emit spikes, the fundamental unit of communication in neural circuits.

In this work we establish this missing theoretical foundation and develop a scaling theory for the relaxation of neural activity to a steady state. To the author’s knowledge, the results obtained follow from the first full theoretic RG analysis of neural populations with leaky integrate-and-fire spiking dynamics. We consider models of both *in vitro* circuits—slices of tissue removed from the brain—and *in vivo* circuits—recordings directly from in-tact brain tissues—and show that they belong to the directed percolation and Ising model universality classes, respectively. We first perform a mean-field analysis of the model to show that it predicts two different types of phase transitions, for which we derive the scaling collapse relations and illustrate the idea of data collapse (Sec. I). We then give and overview of how fluctuations change the mean-field picture and the scaling relations. We show that simulated data of several networks can indeed be collapsed onto universal scaling forms, some yielding mean-field exponents and others anomalous scaling (Sec. III). We end this report by discussing the implications of this work for current theoretical and experimental investigations of collective activity in spiking networks, both near and away from phase transitions (Sec. IV).

## I. SPIKING NETWORK MODEL

We consider a network of N neurons that stochastically fire action potentials, which we refer to as “spikes.” The probability that neuron i fires ${\dot{n}}_{i}(t)dt$ spikes within a small window [t,t+dt] is given by a counting process with expected value $\phi \left(V_{i}(t)\right)dt$, where ϕ(V) is a non-negative firing rate nonlinearity, conditioned on the current value of the membrane potential $V_{i}(t)$. We assume ϕ(V) is the same for all neurons, and for definiteness we will take the counting process to be Poisson or Bernoulli, though the properties of the phase transitions should not depend on this specific choice.

The membrane potential of each neuron obeys leaky dynamics,

(1) $$\tau \frac{dV_{i}}{dt}=-\left(V_{i}-𝓔\right)+\sum_{j=1}^{N}J_{ij}{\dot{n}}_{j}(t),$$

(2) $${\dot{n}}_{i}(t)dt\sim \mathrm{Poiss}\left[\phi \left(V_{i}(t)\right)dt\right]$$

where τ is the membrane time constant, 𝓔 is the equilibrium potential of the neuron in the absence of input, and $J_{ij}\equiv J\left(A_{ij}-\delta_{ij}\right)$ is the weight of the synaptic connection from pre-synaptic neuron j to post-synaptic neuron i. The synaptic weights are characterized by a strength J, and the connections between neurons are encoded by the adjacency matrix $A_{ij}$, which is 1 if neuron j connects onto neuron i and 0 otherwise. We take $J_{ii}=-J$ to be negative in order to implement a “soft” refractory effect, resetting a neuron’s membrane potential by a fixed amount −J after each spike. For simplicity, we model the synaptic input as an instantaneous impulse, referred to as a “pulse coupled” network. We focus on symmetric networks $J_{ij}=J_{ji}$ with a largest real-valued eigenvalue $J{\text{Λ}}_{\text{max}}$ associated with a homogeneous eigenmode, where ${\text{Λ}}_{\text{max}}$ is the maximum eigenvalue of $A_{ij}-\delta_{ij}$. That is, phase transitions in these networks will correspond to pattern formation out of a homogeneous state of activity. While real networks are not homogeneous and symmetric, reciprocal pairs of connections are more common than expected for random networks, and tend to be stronger than uni-directional connections [35]. We interpret our networks as an approximation in which unidi-rectional connections and and variance in the synaptic weights can be neglected. Despite the restriction of a leading homogeneous mode, this encompasses a broad and important class of models and networks, such as bump attractor models in neuroscience [5, 6] and, more generally, diffusion on networks [36].

In this work we consider two nonlinearities corresponding to two types of networks, *in vitro* and *in vivo* networks:

(3) $$\phi (V)=\left\{\begin{array}{cc} \lambda_{0}{\left\lfloor \frac{1}{1+e^{-(V-\theta )/V_{s}}}-r_{0}\right\rfloor }_{+} & ,in\;vitro\;\text{networks}\\ \frac{\lambda_{0}}{1+e^{-(V-\theta )/V_{s}}} & ,in\;vivo\;\text{networks} \end{array},\right.$$

where $V_{s}$ sets the slope of the nonlinearities and $\lambda_{0}$ sets the maximum firing rate of the neurons (equal to $\lambda_{0}\left(1-r_{0}\right)$ for *in vitro* networks and $\lambda_{0}$ for *in vivo* networks). We will choose units such that $V_{s}=\lambda_{0}=1$. The soft threshold θ is the value that the membrane potential needs to exceed in order for a neuron to have an increased probability of firing a spike.

The key difference between the *in vitro* and *in vivo* network nonlinearities is the presence of the rectification in the *in vitro* network nonlinearity, which creates the absorbing state: when $V\leq \theta -\ln\left(r_{0}^{-1}-1\right)$, due to the shift down by $r_{0}$, neurons in an *in vitro* network will not fire. If all neurons’ membrane potentials are below this threshold the network will be completely quiescent and cannot fire spikes without further external input, so this quiescent phase constitutes an absorbing state of the network. In contrast, the instantaneous firing rates of neurons in *in vivo* networks never vanish for any finite input, so there is always some probability that a neuron can fire a spike, even if that probability is small. Other important features of these networks are the saturation of the instantaneous firing rate and the concavity of the nonlinearity. For example, if the nonlinearity is unbounded the network may become unstable for $J>J_{c}$, leading to runaway excitation of the neurons.

In this work we probe the behavior of the network by potentiating or suppressing the membrane potentials of the entire population, and then allowing the network to relax back to a steady state. If the network is tuned to a critical point this relaxation will follow a power law. This procedure would approximate an experimental set-up in which a wide area of neural tissue is optogenetically stimulated or suppressed uniformly. This procedure is similar to experimental setups investigating neural “avalanches”—cascades of neural activity typically originating from a single neuron. However, by potentiating or suppressing the entire network, there is no single neuron that triggers the cascade of activity, allowing us to avoid ambiguity in defining avalanches in the spontaneously active *in vivo* networks, in which it can be unclear whether multiple clusters of activity are really independent events or just non-local parts of a single avalanche.

**FIG. 1.**
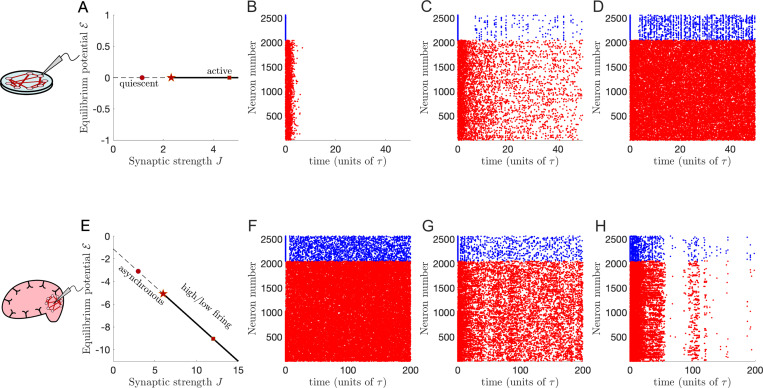
Phase transitions in in vitro versus in vivo neural populations. Neural activity may differ between recorded from tissue maintained or grown in a pitri dish (“*in vitro*”; top row) or recorded directly from neurons in a living organism (“*in vivo*”; bottom row). These differences partly reflect external input: *in vitro* tissue may require experimenter-provided stimulation to maintain the activity of neurons, while *in vivo* neurons are constantly bombarded with input from other brain areas or body systems, leading to spontaneous activity. In both cases qualitative changes in population-level activity may be observed as properties of the network, such as the overall strength of synaptic connections, are modulated. **A.** Phase diagram of an *in vitro* network: if the equilibrium resting potential of the neurons is perturbed (e.g., due to external tonic current input), the network’s firing can be suppressed (𝓔<0) or promoted (𝓔>0). At the equilibrium potential 𝓔=0 (normalized units) the network activity will decay away if the strength of synaptic connections is less than a critical value $J_{c}$. For synaptic strengths $J>J_{c}$ the network activity is self-sustaining. At the critical value $J_{c}$ the activity decays to quiescence, but very slowly. **B.** Example raster plot of spiking activity in a network with subcritical $J=J_{c}/2$ (circle), along the line 𝓔=0, showing a fast decay of activity. **C.** Spiking activity in a network at the approximate critical point $J=J_{c}$ (star), showing slow decay of activity. **D.** Spiking activity in a supercritical network with $J=2J_{c}$ (square), showing sustained activity. **E.** Phase diagram of an *in vivo* network: perturbing the equilibrium resting potential will increase or decrease neural firing. Along a critical line $𝓔={𝓔}_{c}$ (dashed diagonal line) the network will fire asynchronously. For synaptic strengths $J>J_{c}$ there exist states of low or high firing, which the network can transition spontaneously between in finite networks. **F.** Example raster plot of spiking activity in a network with subcritical $J=J_{c}/2$ (circle), along the approximate critical line $𝓔={𝓔}_{c}$, showing asynchronous activity. **G.** Spiking activity in a network at the approximate critical point $J=J_{c}$ (star), showing intermittent high and low spiking activity activity. **H.** Spiking activity in a supercritical network with $J=2J_{c}$ (square), showing apparent transient metastable transitions between high and low firing rate states that are possible in finite-sized network simulations. Excitatory neurons are colored red, inhibitory neurons are colored blue.

In Fig. 1 we display two types of phase transitions this stochastic spiking model can exhibit. The first type involves a transition between a quiescent, inactive state, and a self-sustained active state (Fig. 1A–D). This is an appropriate model for *in vitro* networks, tissue removed from the brain and maintained or cultured in a dish. Such networks receive little-to-no external input other than that which an experimenter provides, hence the possibility for the network to become quiescent if it cannot sustain its activity through recurrent excitation. The second type of transition occurs in spontaneously active networks, exhibiting a transition from asynchronous firing to high and low firing (Fig. 1E–H). This is appropriate as a model of *in vivo* networks, neural tissue that is still part of the brain and receives input from other brain regions. We will first show how to predict these transitions based on a mean-field analysis of the model, and then present the scaling theory that incorporates the effects of stochastic fluctuations that modify the universal quantitative properties that can be measured in experiments.

## II. WIDOM SCALING THEORY

### A. General theory

As seen in the simulations in Fig. 1, the spiking activity undergoes a phase transition at some critical synaptic coupling $J_{c}$ and critical baseline ${𝓔}_{c}$. Because we assume our networks consist of statistically homogeneous neurons, we focus on the dynamics of the population averages of the neurons’ membrane potentials and firing rates $\psi (t)\equiv N^{-1}\sum_{i=1}^{N}\langle V_{i}(t)\rangle$ and $\nu (t)\equiv N^{-1}\sum_{i=1}^{N}\langle {\dot{n}}_{i}(t)\rangle$. Away from the critical point, the population activity decays exponentially to a steady state, while at the critical point the activity decays algebraically—i.e., a power law. However, we can make a stronger statement than this. For networks tuned close to the critical point $\left({𝓔}_{c},J_{c}\right)$ we anticipate that vestiges of scale invariance will lead to scaling collapse: although we are measuring the populations means ψ(t) or ν(t) as a function of three independent parameters, time t, synaptic strength J, and baseline 𝓔, for long times and small enough $J_{c}-J$ and $𝓔-{𝓔}_{c}$ we expect the data can be described by a function of only two combinations of the independent parameters. The generic form of the “Widom scaling relationship” for the population-averaged firing rates is

(4) $$\nu (t)-\nu_{c}\sim t^{-\frac{\beta_{*}}{\nu_{*}z_{*}}}F\left({\left|J_{c}-J\right|}^{\nu_{*}z_{*}}t,\left(𝓔-{𝓔}_{c}\right)t^{\frac{\Delta \nu_{*}}{\nu_{*}z_{*}}}\right),$$

where $\nu_{c}$ is the firing rate at the critical point, F is a scaling function of two arguments, and we have introduced the critical exponents $\beta_{*}$, $\nu_{*}$, $z_{*}$, and $\Delta_{*}$; we give critical exponents subscripts of * to distinguish them from other variables that have similar symbols. The exponents $\nu_{*}$, $z_{*}$, and $\eta_{*}$ are conventionally called the “correlation length,” “dynamic,” and “anomalous” exponents, while $\beta_{*}$ and $\Delta_{*}$ are derived from these exponents by scaling relations we will introduce later. We will retain the name “correlation length exponent” for $\nu_{*}$, even though there may not be a notion of length in arbitrary networks.

Note that the scaling function F is actually multivalued: it can depend on the sign of $J_{c}-J$, $𝓔-{𝓔}_{c}$, and $\nu (t)-\nu_{c}$. This scaling form holds for both the *in vitro* and *in vivo* network models, though the values of the exponents and the scaling function F will differ between the two cases because the two types of networks belong to different universality classes, as we will explain. We derive this scaling form within both the mean-field approximation and our renormalization group analysis in Appendix A.

Eq. (4) tells us that if can determine the correct values of the critical exponents and the critical parameters $\nu_{c}$, $J_{c}$, and ${𝓔}_{c}$, then a plot of $\left(\nu (t)-\nu_{c}\right)t^{\beta_{*}/\nu_{*}z_{*}}$ against ${\left|J_{c}-J\right|}^{\nu_{*}z_{*}}t$ and $\left(𝓔-{𝓔}_{c}\right)t^{\Delta */\nu_{*}z_{*}}$, will “collapse” our three-dimensional dataset onto a two-dimensional surface. In practice, such a collapse is difficult to achieve, and instead one tries to eliminate one of the variables by tuning it to its critical value, and then performing the collapse in the remaining variables, in which case the data should fall onto a one-dimensional curve.

In *in vitro* networks we focus on the case $𝓔={𝓔}_{c}$, and we will collapse firing rates ν(t) for different synaptic strengths using the reduced scaling form

(5) $$\nu (t)\sim {\left|J_{c}-J\right|}^{\beta_{*}}F\left({\left|J_{c}-J\right|}^{\nu_{*}z_{*}}t\right),$$

where $\nu_{c}=0$ and we have pulled a factor of ${\left(t{\left|J_{c}-J\right|}^{\nu_{*}z_{*}}\right)}^{\beta_{*}/\nu_{*}z_{*}}$ out of the scaling function F in order to write the prefactor as ${\left|J_{c}-J\right|}^{\beta_{*}}$.

In our *in vivo* model we will instead consider $J=J_{c}$, for which the scaling form can be reduced to

$$\nu (t)-\nu_{c}\sim t^{-\frac{\beta_{*}}{\nu_{*}z_{*}}}F\left(\left(𝓔-{𝓔}_{c}\right)t^{\frac{\Delta_{*}}{\nu_{*}z_{*}}}\right).$$

The practical difficulty with this scaling form is identifying the critical firing rate $\nu_{c}$. This difficulty can be eliminated by performing two paired experiments: one in which the neurons are potentiated and then allowed to relax to the steady state from above, and another in which the neurons are suppressed and then allowed to relax to the steady state from below, with all other parameters being the same in the two experiments. The scaling function F will differ in these two scenarios (Appendix A), allowing us to obtain a scaling form for the difference in activity:

(6) $$\nu_{+}(t)-\nu_{-}(t)\sim t^{-\frac{\beta_{*}}{\nu *z*}}\bar{F}\left(\left(𝓔-{𝓔}_{c}\right)t^{\frac{\Delta_{*}}{\nu *z_{*}}}\right),$$

where $\bar{F}=F_{+}-F_{-}$, where the sign subscripts correspond to potentiation (+) or suppression (−).

Next, we briefly review the mean-field approximation of the network activity and its predictions for the critical exponents, before highlighting the results of the renormalized scaling theory.

### B. Mean-field scaling theory

The stochastic system defined by Eqs. (1)–(2) cannot be solved in closed form, and understanding the statistical dynamics of these networks has historically been accomplished through simulations and approximate analytic or numerical calculations. A qualitative picture of the dynamics of the model can often be obtained by a mean-field approximation in which fluctuations are neglected, such that $\langle {\dot{n}}_{i}(t)\rangle =\langle \phi \left(V_{i}(t)\right)\rangle \approx \phi \left(\langle V_{i}(t)\rangle \right)$, and solving the resulting deterministic dynamics:

(7) $$\tau \frac{d\langle V_{i}(t)\rangle }{dt}=-\left(\langle V_{i}(t)\rangle -𝓔\right)+\sum_{j=1}^{N}J_{ij}\phi \left(\langle V_{j}(t)\rangle \right).$$

Equations of this form are a cornerstone of theoretical neuroscience [14, 17, 33, 37–39], though often motivated phenomenologically as firing rate models, rather than as the mean-field approximation of a spiking network’s membrane potential dynamics. A wide variety of different types of dynamical behaviors and transitions among behaviors are possible depending on the properties of the connections $J_{ij}$ and nonlinearity ϕ(V) [4, 40], including bump attractors [5, 6, 9, 10], pattern formation in networks of excitatory and inhibitory neurons [11–13], transitions to chaos [14, 15], and avalanche dynamics [7, 8, 16]. In many of these examples, networks admit steady-states for which $d\langle V_{i}\rangle /dt=0$ for all i as t→∞.

Within the mean-field approximation, the dynamics for the population-and-trial-averaged means reduce to

(8) $$\tau \frac{d\psi (t)}{dt}=-(\psi (t)-𝓔)+J{\text{Λ}}_{\text{max}}\nu (t),$$

where ν(t)≈ϕ(ψ(t)). The phase transitions of the network can be characterized by analyzing the dynamics of this one-dimensional system. While Eq. (8) cannot be solved exactly, one can show that there is a continuous bifurcation at $(𝓔,J)=\left({𝓔}_{c},J_{c}\right)$, where the critical baseline ${𝓔}_{c}$ and critical synaptic weight $J_{c}$ are defined by

(9) $${𝓔}_{c}-\psi_{c}+J_{c}{\text{Λ}}_{\text{max}}\phi \left(\psi_{c}\right)=0,$$

(10) $$1-J_{c}{\text{Λ}}_{\text{max}}\phi^{\prime }\left(\psi_{c}\right)=0,$$

in both the *in vitro* and *in vivo* network models. The first condition, Eq. (9), corresponds to the baseline potential 𝓔 and the mean synaptic input to each neuron balancing out so that the membrane potential of the neurons come to rest at $\psi_{c}$, to be determined momentarily. The second condition, Eq. (10), corresponds to this steady state becoming marginally stable: perturbations away from the steady state will not decay away exponentially, nor will they grow exponentially. We expect that when $J>J_{c}$ there will be multiple steady states, so in order for there to be a single marginal state the critical membrane potential $\psi_{c}$ must correspond to the largest value of the gain $\phi^{\prime }(V)$,

(11) $$\psi_{c}=\mathrm{argmax}\phi^{\prime }(V).$$

In the *in vivo* model this corresponds to $\psi_{c}=\theta$, while for the *in vitro* model (with $r_{0}>1/2$), this corresponds to $\psi_{c}=\theta -\ln\left(r_{0}^{-1}-1\right)$, the activation threshold. It follows that $\nu_{c}=0$, ${𝓔}_{c}=\theta -\ln\left(r_{0}^{-1}-1\right)$, and $J_{c}={\left({\text{Λ}}_{\text{max}}r_{0}\left(1-r_{0}\right)\right)}^{-1}$ for the *in vitro* model. For the *in vivo* model we obtain $\nu_{c}=\phi (\theta )$, ${𝓔}_{c}=\theta -2$, and $J_{c}=4/{\text{Λ}}_{\text{max}}$.

In our *in vitro* model the phase transition separates an inactive steady state from an active steady state in which recurrent excitation is strong enough to self-sustain activity without external input, as shown in Fig. 1A–D. In our *in vivo* model the phase transition separates an asynchronous steady state of intermediate firing rates from a bistable state of high- and low-firing rates, as shown in Fig. 1E–H.

At long times and close enough to the critical point we expect ψ(t) to be close to $\psi_{c}$, such that we can expand $\nu (t)=\phi (\psi (t))=\sum_{\ell =0}^{\infty }\frac{\phi^{\left(\ell \right)}\left(\psi_{c}\right)}{\ell !}{\left(\psi (t)-\psi_{c}\right)}^{\ell }$ and truncate at the leading nonlinear order. In our *in vitro* model the leading nonlinear order is ℓ=2, while in the *in vivo* model the leading order is ℓ=3. Plugging this expansion into Eq. (7) and solving for $\psi (t)-\psi_{c}$, and then approximating $\nu (t)\approx \phi \left(\psi_{c}\right)+\phi^{\prime }\left(\psi_{c}\right)\left(\psi (t)-\psi_{c}\right)$ to leading order yields the scaling form (4) with exponents $\beta_{*}=1$ and $\Delta_{*}=2$ in the *in vitro* models [41] and $\beta_{*}=1/2$ and $\Delta_{*}=3/2$ in the *in vivo* model. For both networks $\nu_{*}=1/2$ and $z_{*}=2$.

**FIG. 2.**
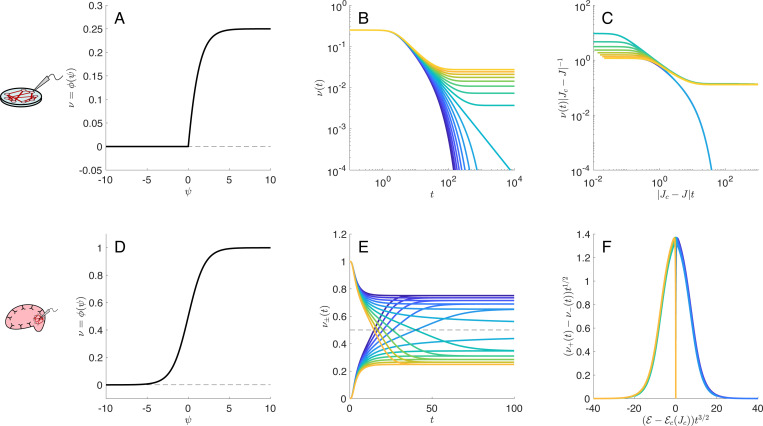
Mean-field behavior of the spiking network for *in vitro* networks (top row) and *in vivo* networks (bottom row). **A,D)** A typical nonlinearity ν=ϕ(ψ) for each of the two network types. In the *in vitro* networks the nonlinearity is rectified, such that the firing rate is zero when a neuron’s membrane potential is negative. In *in vivo* networks the firing rate is never zero—there is always a non-zero, though possibly small, probability of firing. **B,E)** The decay of ν(t), the population- and trial-averaged membrane potential, starting from an initial value of ν(0)≈1 in *in vitro* networks and $\nu_{+}(0)\approx 1$ and $\nu_{-}(0)\approx 0$ in *in vivo* networks. **C,F)** Widom scaling collapses using Eq. (5) for *in vitro* networks and Eq. (6) for *in vivo* networks and the mean-field exponents given in Table I.

We verify that numerical solution of the full mean-field dynamics (Eq. (7)) indeed satisfies the scaling laws when the networks are tuned to their respective critical points. We plot these collapses in Fig. 2 to illustrate the idea of data collapses.

### C. Renormalized scaling theory

While the mean-field approximation generally paints a qualitatively correct picture of phase transitions in a stochastic model, it is well-known that universal quantities like critical exponents or the Widom scaling functions F are often quantitatively incorrect [18], a problem that was ultimately resolved by the development of the renormalization group [42–45].

By developing an RG procedure that can be applied to this spiking network model (detailed in Appendix C), we can capture the effects of stochastic fluctuations on the collective activity of the network. Within our RG approximation scheme, the dynamics of the population averages obey

(12) $$\tau \frac{d\psi (t)}{dt}=-\psi (t)+𝓔+J{\text{Λ}}_{\text{max}}\nu (t),$$

(13) ν(t)=Φ(ψ(t)),

which is similar to Eq. (7) except that the nonlinearity ϕ(ψ) is replaced with an effective nonlinearity Φ(ψ). The key idea behind the RG method presented in Appendix C is that we can compute this effective nonlinearity by iteratively averaging the bare nonlinearity over fluctuations associated with *different eigenmodes* of the synaptic weight matrix $J_{ij}$. In lattices these eigenmodes are simply Fourier bases, which can be parametrized in terms of spatial frequencies (“momenta”) which are traditionally coarse-grained in statistical physics. The eigenvalues of the synaptic weight matrix thus generalize the traditional “momentum-shell” RG approach, though it is not the only possible choice; see also [22, 29, 32, 46].

Near the critical synaptic coupling this effective nonlinearity has the form

(14) $$\text{Φ}(\psi )=\nu_{c}+J^{-1}{\text{Λ}}_{\text{max}}^{-1}\left(\psi -\psi_{c}\right)\;\;\;\;+{\left|J_{c}-J\right|}^{\Delta_{*}}f^{*}\left(\left(\psi -\psi_{c}\right)/{\left|J_{c}-J\right|}^{\beta_{*}}\right)+\ldots ,$$

where $\nu_{c}$ is the critical firing rate of a neuron, $\psi_{c}$ is the critical membrane potential, $f^{*}$ is a universal function, and $\beta_{*}$ and $\Delta_{*}$ are universal critical exponents. Plugging (14) into (12)–(13), the scaling forms (5) and (6) follow with non-trivial values of the critical exponents and scaling functions F.

The scaling function and the critical exponents are characteristics of the “universality class” of a system, which is determined by the (emergent) symmetries of a model. In lattice systems these universality classes are sub-divided by the spatial dimension of the system. i.e., a two-dimensional system will have different critical exponents than a three-dimensional system, despite them both having the same underlying symmetries.

While several notions of dimension have been proposed for complex networks [47], it is not immediately clear which, if any, is the appropriate generalization in the context of critical phenomena. In our RG analysis of the spiking network model (Appendix C), we find that the appropriate generalization of dimension is the *spectral dimension* of the eigenvalue distribution $\rho_{\lambda }(\lambda )$ of the synaptic weight matrix $J_{ij}$, defined by

(15) $$d/2-1\equiv \underset{\lambda \to {\text{Λ}}_{\text{max}}^{-}}{\lim}-\left({\text{Λ}}_{\text{bulk}}-\lambda \right)\frac{d}{d\lambda }\ln\rho_{\lambda }(\lambda ),$$

where ${\text{Λ}}_{\text{bulk}}$ is the maximum eigenvalue of the continuous part of the eigenvalue spectrum and ${\text{Λ}}_{\text{max}}$ is the maximum eigenvalue of the network. If ${\text{Λ}}_{\text{bulk}}={\text{Λ}}_{\text{max}}$, then the spectrum is continuous near the maximum eigenvalue and $\rho_{\lambda }(\lambda )\sim {\left({\text{Λ}}_{\text{max}}-\lambda \right)}^{d/2-1}$; the definition of d here is chosen so that it matches the spatial dimension when the network is a hypercubic lattice with periodic boundary conditions. If ${\text{Λ}}_{\text{bulk}}\neq {\text{Λ}}_{\text{max}}$, then the largest eigenvalue is an outlier and d diverges—this will be relevant in the case of random regular networks we study in Sec. III. The spectral dimension has been identified as the relevant definition of dimension in other work investigating critical dynamics of, e.g., Ising-like models or random walks on networks [48–50].

We can further show that the *in vitro* and *in vivo* models belong to different universality classes. The *in vitro* model belongs to the “directed percolation” universality class, a ubiquitous non-equilibrium universality class that describes the transition between extinction of activity and self-sustained activity. The directed percolation universality class is characterized by an emergent “rapidity symmetry” that relates the magnitude of the membrane potential to fluctuations in the spiking activity, and three independent exponents, the correlation length exponent $\nu_{*}$, the dynamic exponent $z_{*}$, and the anomalous exponent $\eta_{*}$. The other critical exponents can be expressed as $\beta_{*}=\frac{\nu_{*}}{2}\left(d+\eta_{*}\right)$ and $\Delta_{*}=\frac{\nu_{*}}{2}\left(d+2z_{*}-\eta_{*}\right)$ [51]. The directed percolation universality class has an “upper critical dimension” of d=4, which means that networks with spectral dimension above this value will display mean-field scaling. Networks with ${\text{Λ}}_{\text{bulk}}\neq {\text{Λ}}_{\text{max}}$ have d=∞, and are above the upper critical dimension. The values of these critical exponents in lattice systems and mean-field are summarized in Table I.

Turning to the *in vivo* model, our RG analysis predicts that the model belongs to the Ising model universality class, which describes transitions from a disordered state to ordered states related by an inversion symmetry. In the spiking network the two ordered states are the high and low firing rate states, and the inversion symmetry implies that close to the phase transition the distribution of fluctuations around the means of the high and low firing rates are identical. The universality class of the non-equilibrium Ising model is characterized by the correlation length exponent $\nu_{*}$, the dynamic exponent $z_{*}$, and anomalous exponent $\eta_{*}$, with $\beta_{*}=\frac{\nu_{*}}{2}\left(d-2+\eta_{*}\right)$ and $\Delta_{*}=\frac{\nu_{*}}{2}\left(d+2-\eta_{*}\right)$. Like the directed percolation universality class, the Ising model has an upper critical dimension of d=4, above which the mean-field approximation predicts the correct scaling. The values of the critical exponents for two- and three-dimensional lattices and mean-field are given in Table I.

The values of the critical exponents are not necessarily the same for neurons arranged in lattices and complex networks, even if they have the same spectral dimension d. Our RG scheme, described in Appendix C, does predict that lattices and networks with the same spectral dimension will have the same exponents, but we expect this to be only true approximately. Being the first application of the RG to a spiking population model, our method does not capture the effects of the eigenmode structure of the synaptic weight matrix on the critical exponents, which could impact the values of $\eta_{*}$ and $z_{*}$ in particular, which our method predicts to have the mean-field values $\eta_{*}=0$ and $z_{*}=2$. However, our method does predict anomalous values of $\nu_{*}$, $\beta_{*}$, and $\Delta_{*}$. This said, because our RG scheme predicts the universality classes of the *in vitro* and *in vivo* networks, we can use the full set of anomalous exponents from d-dimensional lattices as a starting point for the scaling collapses we perform on our simulated data, allowing us to estimate potential discrepancies between lattices and networks with the same spectral dimension.

Finally, within our RG approximation we can also analytically compute the asymptotic tails of the scaling functions F appearing in the Widom scaling forms. For *in vitro* networks we find

(16) $$F(x)\sim \left\{\begin{array}{cc} \exp\left(-C_{<}x\right), & J<J_{c}\\ y_{\infty }+B_{>}\exp\left(-C_{>}x\right), & J>J_{c} \end{array},\right.$$

where $x\propto {\left|J_{c}-J\right|}^{\nu_{*}z_{*}}t$ and the constants $C_{<}$, $C_{>}$, $B_{>}$, and $y_{\infty }$ are non-universal constants. In *in vivo* networks we find that the tails of the scaling function (6) obey

(17) $$\bar{F}(x)\sim x^{\frac{\beta_{*}}{\Delta *}}\exp\left(-Cx^{1-\frac{\beta_{*}}{\Delta *}}\right),$$

where $x\propto \left(𝓔-{𝓔}_{c}\right)t^{\frac{\Delta_{*}}{\nu^{*}z_{*}}}$ and C is another non-universal constant. Up to the non-universal constants, we show in the next section that not only can we collapse simulated activity data, the collapses agree well with the predicted scaling functions.

## III. SCALING ANALYSES OF SIMULATED DATA

To validate our scaling theory, we first show that simulated data from neurons arranged on 2- and 3-dimensional lattices with nearest-neighbor excitatory connections are indeed collapsed using the directed percolation or Ising model critical exponents listed in Table I.

We then investigate scaling in networks in which excitatory neurons are sparsely connected with fixed degree and inhibitory neurons, if present, provide broad global inhibition. Our primary goal is to verify that the universality classes of the *in vitro* and *in vivo* models are consistent with the directed percolation and Ising universality classes, respectively. We do not seek to obtain high precision estimates of the exponents competitive, instead devoting our computational resources to estimating the exponents on several network types.

### A. Excitatory lattices

**FIG. 3.**
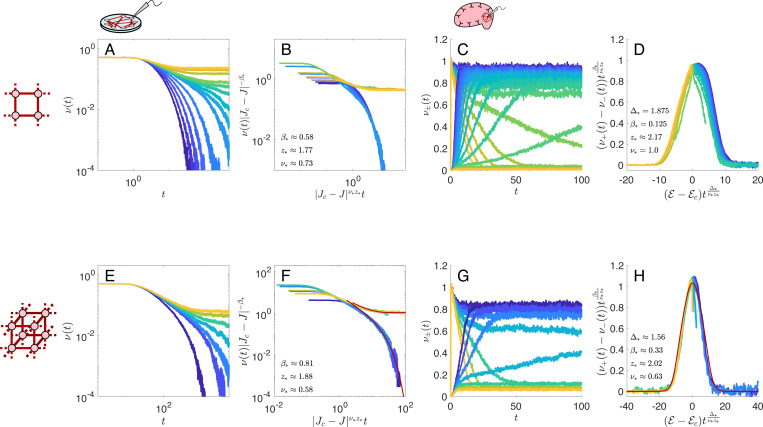
Widom scaling collapses for simulated activity on excitatory lattices. Top row (**A**-**D**): d=2. Bottom row (**E**-**H**): d=3. **A, E.** Population-and-trial-averaged spike trains ν(t) versus time in *in vitro* networks as the synaptic strength J is tuned from subcritical ($J<J_{c}$, blue curves) to supercritical ($J>J_{c}$, green-gold curves). **B, F.** Widom scaling collapse of the data using Eq. (5). Data below and above $J_{c}$ collapse onto different curves, with tails given by Eq. (16). **C, G.** Population-and-trial-averaged spike trains versus time in *in vivo* networks, starting from a high firing rate initial condition $\nu_{+}(0)\approx 1$ and a low firing rate initial condition $\nu_{-}(0)\approx 0$. Curves correspond to equilibrium potentials $𝓔>{𝓔}_{c}$ (green-gold curves) to $𝓔<{𝓔}_{c}$ (blue curves). **D, H.** Widom scaling collapse of $\nu_{+}(t)-\nu_{-}(t)$ according to Eq. (6), with tails given by Eq. (17). The critical exponents used to collapse the data, inset in each collapse, are the known values of the critical exponents for the directed percolation (DP) and Ising model (IM) universality classes, given in Table I.

As shown in Fig. 3A–B, simulated data from *in vitro* models can be collapsed using the direction percolation critical exponents and the scaling form (5), and simulated data from *in vivo* models can be collapsed using the Ising critical exponents and the scaling form (6).

In our *in vitro* networks the critical synaptic weights are $J_{c}\approx 1.56$ in d=2 and $J_{c}\approx 0.821$ in d=3, while in the spontaneous networks the critical parameters are $\left({𝓔}_{c},J_{c}\right)\approx (-4.27,3.3)$ in d=2 and $\left({𝓔}_{c},J_{c}\right)\approx (-2.79,1.165)$ in d=3.

### B. Sparse excitation and dense inhibition

We now consider networks with slightly more realistic features. Cortical circuits consist of two broad cell types: excitatory and inhibitory. Excitatory cells are typically thought to be the “principal neurons” whose activity is the neural realization of computations within cortical circuitry, while inhibitory cells are often “interneurons” that serve these computations indirectly by regulating the activity of the principal neurons. In cortex, excitatory neurons have been found to make sparse connections to other excitatory neurons [56], instead influencing each other through the densely connected inhibitory interneurons [57]. We therefore consider a network in which excitatory neurons make sparse connections to one another; specifically, we will model excitatory-excitatory connections using random regular graphs in which every neuron makes a fixed number of synaptic connections k but the pairs of neurons connected are randomly chosen, independent of any spatial organization of the network. The remaining connections in the network are dense; for simplicity we take the connections from excitatory to inhibitory cells, as well as inhibitory-to-inhibitory or inhibitory-to-excitatory, to be all-to-all connected.

**FIG. 4.**
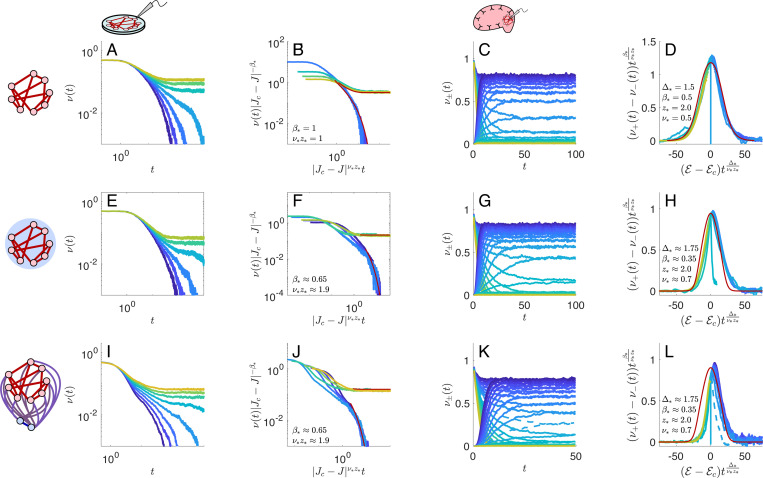
Widom scaling collapses for simulated activity on networks with random regular excitatory-excitatory connections. All excitatory neurons make k=3 excitatory connections to other neurons. Top row: Simulation results for purely excitatory networks. **A.** Decay of the population-averaged spiking activity in an absorbing state network for several values of coupling strength J, and **B.** its corresponding data collapse using mean-field predictions for the critical exponents. **C.** Decay of the population-averaged spiking activity in a spontaneously active network for several values of the input current 𝓔, and **D.** its corresponding data collapse using mean-field predictions for the critical exponents. Middle row: Simulation results for an excitatory population with effective inhibitory connections between neurons. **E-F** and **G-H** are the same as **A-B** and **C-D**, but using anomalous values of the critical exponents. Bottom: Simulation results for a model of separate excitatory and inhibitory populations that reduces to the effective model (Appendix B). **I-J** and **K-L** are the same as **E-F** and **G-H**, using the same values of the anomalous exponents. In the absorbing state collapses (second column), the analytically estimated asymptotic Widom scaling forms Eq. (16) are plotted in red, scaled by non-universal factors to match the data. Similarly for the spontaneous network collapses (fourth column) using Eqs. (6) and (17); see also Appendix A.

First, it is useful to consider what happens in networks without inhibition, for which we need only consider the excitatory neurons arranged in a random regular network. If the synaptic strength of each connection is J and each neuron has a refractory self-input of strength −J, then the eigenvalue distribution of the synaptic weight matrix has a maximum eigenvalue $J{\text{Λ}}_{\text{max}}=J(k-1)$ associated with the homogeneous mode. This eigenvalue is an outlier. The bulk spectrum of the synaptic weight matrix is given by the McKay law [58] in the N→∞ limit (modified to include the self-coupling and normalized by J),

$$\rho_{\lambda }(\lambda )=\frac{2k}{\pi }\frac{\sqrt{4(k-1)-{\left(\lambda +1\right)}^{2}}}{4k^{2}-4{\left(\lambda +1\right)}^{2}};$$

where $\lambda \in [-2\sqrt{k-1}-1,2\sqrt{k-1}-1]$. Note that the bulk spectrum has spectral dimension d=3, independent of the degree k. The contribution of the single outlier eigenvalue contributes negligibly to the effective nonlinearity Φ(ψ), but it nonetheless controls the phase transition because it renders the spectral dimension to be d=∞ (Eq. (15)). We therefore expect that the excitatory random regular network will exhibit mean-field critical exponents; we confirm this in the scaling collapses of both *in vitro* and *in vivo* networks, shown in Fig. 4. We find that the transitions occur approximately at $J_{c}\approx 2.17$ in *in vitro* networks and $\left({𝓔}_{c},J_{c}\right)\approx (-3.5,3.75)$ in *in vivo* networks.

Next, we consider what happens when we turn on the inhibitory connections. Rather than analyzing the full EI population, it is useful to first consider an *effective* network model consisting of excitatory neurons that excite their random regular neighbors while inhibiting all other neurons in the network. This effective model is a formal reduction of a full population model with explicit excitatory and inhibitory populations; see Appendix B. Suppose the global inhibitory connections have strength −Jc/N, where N is the number of excitatory neurons. These inhibitory connections will shift the location of ${\text{Λ}}_{\text{max}}$ from k−1 to k−1−c, without affecting any other eigenmodes of the network because they are orthogonal to the homogeneous mode. There is then a critical value of $c=k-2\sqrt{k-1}$ for which the maximum eigenvalue is moved to the location of the bulk eigenvalue ${\text{Λ}}_{\text{bulk}}=2\sqrt{k-1}-1$, closing the gap between the bulk spectrum and the outlier. We then expect the effective dimension to be d=3, and the network may exhibit anomalous scaling instead of mean-field scaling. We verify this for networks with k=3 for both the effective EI network and networks with explicit inhibitory neurons.

In the case of the effective EI networks, we find $J_{c}\approx 2.32$ with approximate exponents $\beta_{*}\approx 0.65$ and $\nu_{*}z_{*}\approx 1.9$ in *in vitro* networks, and $J_{c}\approx 6.0$, $\beta_{*}\approx 0.35$, $\nu_{*}\approx 0.7$, $z_{*}\approx 2$ in *in vivo* networks. The same exponents with $J_{c}\approx 2.39$ and $J_{c}\approx 6.0$ in *in vitro* and *in vivo* networks, respectively, produce collapses in simulations with explicit excitatory and inhibitory populations (Fig. 4). While these estimates are not especially precise, the *in vitro* exponents differ enough from the exponents in the d=3 lattices to suggest that the network structure does have an influence on the critical exponents, and hence the universality class of the networks may differ from the 3-dimensional lattices, although these universality classes are still in some sense close.

We see that the collapses for the effective and explicit EI networks are asymmetrically skewed compared to the analytically predicted Widom scaling forms for the d=3 Ising universality class, which match the d=3 lattice well. Similar asymmetries have been observed in the scaling collapses of mean neural avalanche shapes, and recent modeling work has shown that inhibitory neurons play a role in producing these asymmetries [59]. It is not clear what the origin of this asymmetry is. Potentially it is a finite-size effect that will weaken in simulations of much larger networks. A surface plot of the firing rates versus J and 𝓔 does not show a sharp jump, compared to the lattices, suggesting that finite size effects may be softening the transition. For instance, the dashed curve in Fig. 4K–L corresponds to a value of 𝓔 that is close to the estimated critical ${𝓔}_{c}$, and does not collapse well in our current simulations, but may be more sharply separated from the critical point in larger networks. Alternately, the asymmetries could be driven by subleading corrections to scaling or contributions from the network eigenmodes that are not captured by our current RG scheme.

Next, we perform a brief investigation of networks with degree k>3. There is a common folk wisdom that mean-field theory becomes accurate in high dimensional lattices because of the increased number of neighbors each unit interacts with. However, the spectral dimension of random regular networks does not depend on the number of neighbors k. We might therefore wonder whether the critical exponents will remain close to the values predicted by our scaling theory, or if at a sufficiently large k we see a reversion to mean-field behavior.

We find that networks with k≥4 and global inhibition strength $c=k-2\sqrt{k-1}$ exhibit mean-field scaling. However, the observed critical strength $J_{c}$ is lower than the mean-field prediction, in contrast to our other simulations. This suggests that the global inhibition may be too large. The mismatch most likely originates in our RG scheme’s independence of the eigenmode structure of the network, and a higher order approximation scheme is required to identify the precise value of global inhibition at which anomalous scaling is observed for k>3.

We may therefore wonder how robust the anomlous scaling of the k=3 network is to perturbations in the number of connections each neuron makes. So far, we have considered networks in which the number of connections each neuron makes is the same for all neurons. If we instead consider networks with a fraction f of neurons that make k=3 synaptic connections and a fraction 1−f of neurons that make k=4 synaptic connections, we can investigate between which fractions we observe a transition from anomalous to mean-field scaling.

The excitatory synaptic connections between neurons are $J_{ij}=J\left(A_{ij}-\left(k_{i}-2\right)\delta_{ij}-c/N\right)$, where $A_{ij}$ is the adjacency matrix of the excitatory connections, formed by randomly paring up synapses of fN degree 3 neurons and (1−f)N degree 4 neurons (and rejecting networks with self-connections or multiple synapses between a single pair of neurons); i.e., this is a configuration model [60]. $k_{i}$ is the degree of neuron i, and c is again the all-to-all inhibitory weight chosen to move the leading eigenvalue of $A_{ij}-\left(k_{i}-2\right)\delta_{ij}$ to the edge of the bulk spectrum. Although we do not have a closed form for the spectrum of this weight matrix, the limiting cases f=1 and f=0—random regular networks of degrees 3 and 4, respectively—have spectral dimension d=3, and so we expect the mixed network does as well. We find that at 1−f=10% neurons with degree 4 the anomalous scaling persists, while at 1−f=20% we again obtain mean-field scaling.

While we have not ruled out that there is a different value of global inhibition c that can achieve anomalous scaling in networks with mixed connectivity, our results suggest a possible new mechanism that could explain apparently contradictory observations of mean-field versus anomalous scaling in neural avalanche data [8, 27, 28], with the observed scaling depending on the balance of excitatory sparsity and broad inhibition.

## IV. DISCUSSION

In this work we have shown that stochastic spiking networks with symmetric connections and homogeneous steady states can undergo at least two types of phase transitions as the strength of their synaptic connections J and baseline potentials 𝓔 are tuned: i) an inactive-to-active transition between extinction and self-sustained activity, appropriate as an *in vitro* model of neural tissue, and ii) a transition from a single asynchronous state of activity to high or low firing rate states, appropriate as a model of *in vivo* neural tissue.

Using both a mean-field approximation and a renormalization group analysis of the spiking network model, we developed a Widom scaling theory to show that the *in vitro* network models belong to the directed percolation universality class, while the *in vivo* network models belong to the Ising universality class. These universality classes are subdivided by “dimension,” which we identified as the spectral dimension of the synaptic weight matrix. If the largest eigenvalue of this spectrum is an outlier, then the spectral dimension is infinite, and we find the mean-field predictions of the critical exponents are correct. However, if the largest eigenvalue is at the edge of the continuous part of the spectrum, then d is finite and anomalous scaling may be observed.

This is, to the author’s knowledge, the first renormalization group analysis of a leaky integrate-and-fire model. While previous work modeling phase transitions in neural populations have used Ising-like models or chemical reaction networks of active or inactive neurons, these are phenomenological models of neural activity. Similarly, while other work has investigated the non-perturbative renormalization group in neuroscience contexts, it has been applied only to calculating correlation and response functions in firing rate models [33], and exploring possible equivalences between the RG and neural sensory coding work [46]. This work establishes formally that spiking populations are in the Ising model or directed percolation universality classes.

The value of performing renormalization group calculations on spiking network models is that these calculations help clarify what features of neurons and their connectivity shape the critical exponents measured in data. Experimental recordings of neural avalanches—cascades of neural activity triggered that propagate through neural circuitry [7, 8, 16, 25]—often seem to support mean-field exponents, but deviations have also been reported [8], and the origin of these deviations remains the subject of much debate. Some reports suggest deviations could be the effects of subsampled recordings of neurons in space or time [61, 62], while RG analysis of firing rate models suggest the cause could also be logarithmic corrections to critical exponents in networks at their upper critical dimension [34]. Similarly, the “phenomenological RG” method developed by Ref. [22] motivates exponent relations based on analogies with lattice systems, and find anomalous scaling for the dynamical exponents $z_{*}$, albeit with values on the order of 0.16 ∼ 0.3, much smaller than $z_{*}\approx 2$ predicted by the directed percolation or Ising model universality classes. Similar exponent values are obtained by Ref. [63] in rat visual cortex. Using our foundational RG theory of spiking networks to understand the phenomenolgoical RG method could yield insight into these unexpectedly small dynamical exponents.

In general, having a firm theoretical understanding of what properties influence critical exponents will not only aid in disambiguating genuine deviations from mean-field theory versus estimates are skewed by subsampling problems, but could also reveal mechanisms by which a system that appears to be in a mean-field universality class could be tuned toward anomalous behavior.

For instance, both the directed percolation and Ising model mean-field universality classes make the same predictions for avalanche exponents, but in lower dimensions the exponents and even the exponent relations differ. In our excitatory-inhibitory network of sparse excitatory connections that interact through dense inhibitory connections, we found that the inhibition could tune the network to a critical point with anomalous exponents for both the directed percolation and Ising universality classes. One could imagine a future closed-loop experimental paradigm in which excitatory neurons are inhibited with wide-field optogenetic stimulation, where the strength of that stimulus depends on the recorded neural activity. Depending on the properties of the excitatory connections (e.g., the degree of sparsity), this could drive a transition toward a anomalous critical state, distinguishing between different mean-field universality classes.

Several other mechanisms have been proposed as possible causes of mean-field or anomalous scaling. Ref. [34] introduced a firing rate model in which nonlinearities in the membrane dynamics cancel out nonlinearities in the mean synaptic input, resulting in a new universality class with upper critical dimension d=2. At this upper critical dimension the critical exponents differ from the mean-field scaling by logarithmic corrections that can depend on the distance to the critical point, introducing apparent anomalous scaling.

Another mechanism well-known to change critical exponents is heterogeneity in network properties, often dubbed “disorder” in the statistical physics literature. In this work we considered only heterogeneity in the pairs of neurons connected, and observed that it potentially alters critical exponents compared to lattice networks with the same effective dimension. We assume our networks can be interpreted as the average connectivity in networks with weak variability that can be neglected. Strong heterogeneity in the synaptic weights, however, can have a variety of possible effects. It can smear out a transition, effectively destroying it, drive the system to a different “strong disorder” universality class with different critical exponents, or lead to anomalously slow temporal scaling $\exp\left(-t^{a}\right)$ or $t^{-b}$ for non-universal exponents a and b due to the existence of “rare regions” of the network that happen to be close to the critical point of the non-disordered system [64–66].

Because we have shown that the spiking network belongs to the directed percolation or Ising universality classes, we can leverage past work on other systems in the directed percolation [67–69] or Ising classes [70, 71] to motivate hypotheses for how heterogeneity might impact criticality in spiking networks. For example, Ising models with heterogeneity in properties analogous to the baseline potential 𝓔 and synaptic strength J are thought to belong to the random field Ising model universality class [70, 71], for which renormalized scaling theories have been derived using extensions of the methods we use to obtain the results reported in this work [72–76].

Heterogeneity in synaptic weights drives perhaps the most well known example of a phase transition in theoretical neuroscience, the celebrated transition to chaos in the Sompolinsky-Crisanti-Sommers model [14, 37], a firing rate model with random recurrent synaptic connections that can be interpreted as the mean-field theory of the spiking network studied in this work. This model and its many descendants have been extensively studied using methods from dynamic mean-field theory, and have become a cornerstone of theoretical neuroscience. The transition to chaos is not in the directed percolation or Ising model universality classes, owing in part to the fact that the synaptic connections have zero mean and are not symmetric. An important direction of future work is to extend our RG scaling theory to such networks and investigate whether stochastic spiking generates anomalous scaling in the transition to chaos. While fluctuations have been added to this family of models by adding fluctuating external currents or Poisson inputs with rates matching the firing rate of the networks, mimicking the effect of spike fluctuations [77, 78], and suggest that mean-field scaling persists in the presence of fluctuations, it remains an open question whether this is robust to adding structure to the synaptic connections, such as occurs in the networks studied in this work.

This said, all cases we have considered so far assume fixed synaptic connectivity. This is a reasonable assumption on timescales comparable to the millisecond scales of neural activity. However, on longer timescales these connections can be modified by synaptic plasticity, in which neural activity drives strengthening or weakening of synaptic connections. Including synaptic plasticity in the stochastic spiking model couples the neural dynamics with their synaptic tuning parameters. Ref. [79] investigated plasticity dynamics in a Sompolinsky-Crisanti-Sommers firing rate model using a dynamic mean-field approach, but this phenomena has yet to be explored through using RG scaling analyses. It is often hypothesized that synaptic plasticity and homeostasis will lead to *self-organized criticality*, with some simulation results supporting the possibility in simple models [80]. This would imply that the synaptic strengths and baseline potentials of the networks are self-regulated towards their critical values over time [81–83]. Understanding the impact of disorder and synaptic plasticity on critical properties is crucial for interpreting neural data in the context of criticality, and the approach presented here establishes an important first step toward analyzing critical phenomena in spiking network models with heterogeneous features.

## Supplementary Material

Supplement 1

## Figures and Tables

**FIG. 5.**
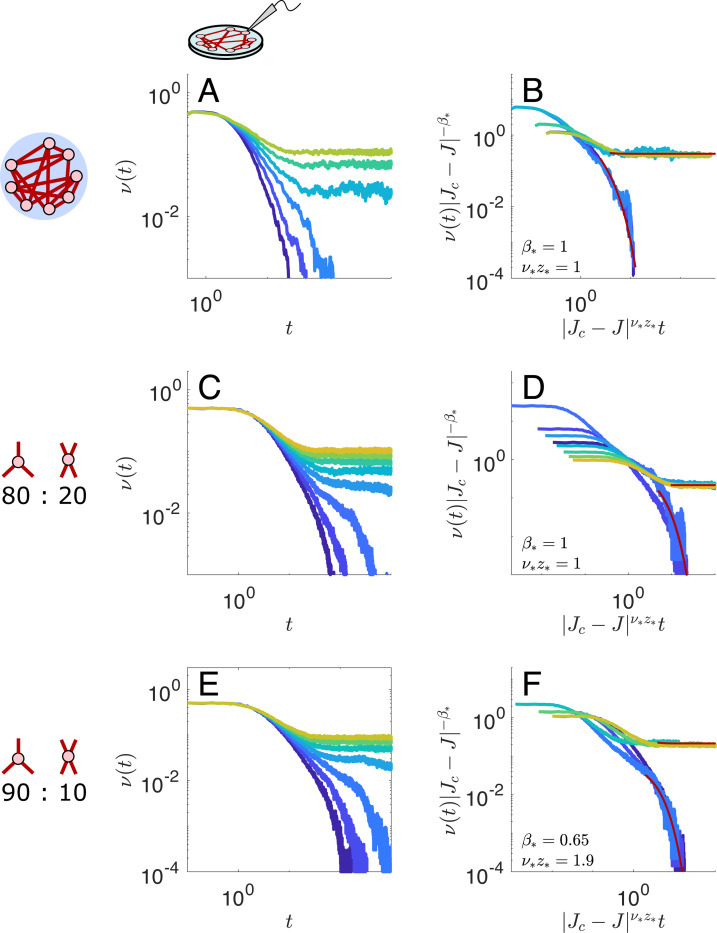
Mean-field versus anomalous scaling in Excitatory-Inhibitory networks of varying degree. **A-B.** Simulated activity in an effective EI network with degree 4 random regular excitatory-excitatory connections, and **B.** its corresponding scaling collapse using mean-field exponents. The phase transition occurs at a value of $J_{c}\approx 1.38$ that is less than the mean-field prediction $J_{c}=4/(2\sqrt{3}-1)\approx 1.62$, whereas in our other cases our RG analysis predicts that $J_{c}$ is larger than the mean-field prediction. **C-D.** Effective EI network with 20% degree 4 and 80% degree 3 excitatory-excitatory connections. The phase transition occurs at $J_{c}\approx 1.98$, less than the mean-field prediction $J_{c}\approx 2.26$. The data can be collapsed using mean-field exponents. **E-F.** Effective EI network with 10% degree 4 and 90% degree 3 excitatory-excitatory connections. The estimated critical coupling is $J_{c}\approx 2.2$, comparable to the mean-field prediction of $J_{c}\approx 2.23$. The data, however, collapses using the same anomalous exponents as the pure degree 3 effective EI network.

**TABLE I.** Critical exponents for the Directed percolation and Ising model universality classes on lattices of d=2 and 3 dimensions, compared to the mean-field (MF) prediction. Values are given to the hundredths place; more accurate estimates are available in the cited references.

	Directed percolation [52]			Ising model		
	
	d=2	d=3	MF	d=2[18]	d=3[53]	MF
$\nu_{*}$	0.73	0.58	1/2	1	0.63	1/2
$z_{*}$	1.77	1.89	2	2.17 [54]	2.02 [55]	2
$\eta_{*}$	−0.41^†^	−0.17^†^	0	1/4	0.036	0
$\beta_{*}$	0.28	0.82	1	1/8	0.33	1/2
$\Delta_{*}$	1.87^†^	1.91^†^	2	15/8	1.56	3/2

† Derived exponents using the scaling relations $\beta^{*}=\frac{\nu_{*}}{2}\left(d+\eta_{*}\right)$ and $\Delta_{*}=\frac{\nu_{*}}{2}\left(d+2z_{*}-\eta_{*}\right)$ for the directed percolation universality class [51].
