# Supplementary material for: Phase transitions in in vivo or in vitro populations of spiking neurons belong to different universality classes
Source: ArXiv. 2025 May 13:arXiv:2301.09600v4. Preprint. [Version 4] (PMC12133079)
Supplement: Supplement 1 [file NIHPP2301.09600v4-supplement-1.pdf]

Appendix A: Derivation of the Widom scaling forms

Here we derive the Widom scaling forms (5) and (6), both in the mean-field approximation and using the results of our renormalization group analysis (described in Appendix C).

Mean-field scaling forms

In vitro *networks*

In our *in vitro* network models we set the baseline to its critical value, which we may take to be $\mathcal{E}_c = 0$, so we need only derive the scaling form as the synaptic weight J is changed. We choose parameter conventions so that the steady-state value of the membrane potential is $\psi_c = 0$, and we assume that $\phi''(0^+) < 0$. Then, close to $\psi(t) = 0$ the mean-field dynamics (7) reduce to

$$\tau \frac{d\psi(t)}{dt} = -(1 - J\Lambda_{\max}\phi'(0))\psi(t) + \frac{J\Lambda_{\max}}{2!}\phi''(0)\psi(t)^2 + \ldots .$$

Neglecting the higher order terms, we can solve this by separation of variables, giving the implicit solution

$$\int_{\psi(0)}^{\psi(t)} \frac{dy}{-(1 - J\Lambda_{\max}\phi'(0))y + \frac{J\Lambda_{\max}}{2!}\phi''(0)y^2} = \frac{t}{\tau}.$$

Although this integral can be evaluated exactly, to derive the scaling form it is useful to work with the integral form. Comparing to Eq. (5), we want a relationship between $(J_c - J)\psi(t)$ and $(J_c - J)t$, where $J_c - J \propto 1 - J\Lambda_{\max}\phi'(0)$. This motivates a change of variables $\hat{y} = y\xi_\tau$, where $\xi_\tau^{-1} \equiv (1 - J\Lambda_{\max}\phi'(0))/\tau$, which gives

$$\int_{\psi(0)\xi_\tau}^{\psi(t)\xi_\tau} \frac{d\hat{y}}{-\hat{y} + \frac{J\Lambda_{\max}}{2\tau}\phi''(0)\hat{y}^2} = \frac{t}{\xi_\tau}.$$

If we assume that $\psi(0)\xi_\tau \gg 1$, such that we can approximate the lower limit as ∞ if $\xi_\tau > 0$ or $-\infty$ if $\xi_\tau < 0$, then the left hand side is a function of $\psi(t)\xi_\tau$ and the right-hand-side is t/ξ_τ. Assuming that this function can be inverted gives a functional relationship that satisfies the scaling form (5).

We can check this against the exact solution,

$$\psi(t) = \frac{\psi(0)e^{-t/\xi_\tau}}{1 + \frac{J\Lambda_{\max}\xi_\tau|\phi''(0)|}{2\tau}\psi(0)(1 - e^{-t/\xi_\tau})},$$

If $0 < \xi_\tau < \infty$ and $\xi_\tau\psi(0) \gg 1$, then for long times this reduces to $\psi(t) \approx \frac{2\tau}{J\Lambda_{\max}|\phi''(0)|}\xi_\tau^{-1}\exp(-t/\xi_\tau)$, which reveals that $F(x) \propto \exp(-x)$.

If $-\infty < \xi_\tau < 0$, then for long times $\psi(t) \approx$

$\frac{2\tau}{J\Lambda_{\max}|\phi''(0)|}|\xi_\tau^{-1}|(1-\exp(-t/|\xi_\tau|))$, giving $F(x) \propto 1 - \exp(-x)$.

Finally, if $\xi_\tau \to \infty$ we find $\psi(t) \approx \frac{2\tau}{J_c\Lambda_{\max}|\phi''(0)|}t^{-1}$, the expected power-law decay.

In vivo *networks*

We will work at the critical synaptic weight $J = J_c$, where $J_c = (\Lambda_{\max}\phi'(\psi_c))^{-1}$, where $\psi_c = \theta$ is the potential for which $\phi''(V) = 0$. We expand the nonlinearity in the mean-field equation (7) around θ, yielding

$$\tau\frac{d}{dt}(\psi(t)-\theta) = \mathcal{E} - \mathcal{E}_c + \frac{J\Lambda_{\max}}{3!}\phi^{(3)}(\theta)(\psi(t)-\theta)^3 + \dots$$

where the linear term has vanished because $J = J_c$ and we defined $\mathcal{E}_c \equiv \theta - J_c\Lambda_{\max}\phi(\theta)$. Solving the equation implicitly, with $u(t) = \psi(t) - \theta$,

$$\int_{u(0)}^{u(t)} \frac{dy}{\mathcal{E} - \mathcal{E}_c + \frac{J\Lambda_{\max}}{3!}\phi^{(3)}(\theta)\hat{y}^3} = \frac{t}{\tau}$$

In the mean-field approximation we expect the scaling variables to be $(\psi(t)-\theta)t^{1/2}$ and $(\mathcal{E}-\mathcal{E}_c)t^{3/2}$. This motivates a change of variables $\hat{y} = y(t/\tau)^{1/2}$, which gives

$$\int_{u(0)(t/\tau)^{1/2}}^{u(t)(t/\tau)^{1/2}} \frac{d\hat{y}}{(\mathcal{E}-\mathcal{E}_c)(t/\tau)^{3/2} - \frac{J\Lambda_{\max}}{3!}|\phi^{(3)}(\theta)|\hat{y}^3} = 1$$

Assuming that $|u(0)|(t/\tau)^{1/2} \gg 1$, we can approximate the lower limit as $-\infty$ if $u(0) < 0$ and $+\infty$ if $u(0) > 0$. Note that although the value of $\psi(0)$ will not contribute to the asymptotic scaling form, its sign does determine the relevant branch of the evaluation of the integral, which will yield two different scaling forms. The left-hand-side is then a function of both $(\psi(t)-\theta)(t/\tau)^{1/2}$ and $(\mathcal{E}-\mathcal{E}_c)(t/\tau)^{3/2}$, which is equal to a constant, imposing a functional relationship between the two scaling variables. The scaling forms will both be of the form $(\psi(t)-\theta)(t/\tau)^{1/2} = F_\pm((\mathcal{E}-\mathcal{E}_c)(t/\tau)^{3/2})$. Once we obtain the scaling forms numerically, we can subtract them to get a single scaling form for $\psi_+(t) - \psi_-(t)$, which will be of the form (6)

Renormalized scaling theory

The derivations of the Widom scaling forms using the effective nonlinearity $\Phi(\psi)$ are similar to the mean-field calculations, with some additional subtleties to manage.

Plugging the expansion of $\Phi(\psi)$, Eq. (14), into the homogeneous dynamics of the trial-averaged means, Eqs. (12)-(13), and keeping only the leading order nonlinear contribution to the effective nonlinearity:

$$\begin{aligned}\tau\frac{d\psi}{dt} &= -\psi + \mathcal{E} + J\Lambda_{\max}\Phi(\psi)\\ &= \mathcal{E} - \mathcal{E}_c\\ &\quad + J\Lambda_{\max}|J_c - J|^{\Delta_*}\\ &\quad\quad \times f^*\left((\psi-\psi_c)|J_c-J|^{-\beta_*}\right);\end{aligned}$$

where we define $\mathcal{E}_c \equiv \psi_c + \Lambda_{\max}\nu_c$. Next, we consider our two specific network cases.

In vitro *networks*

For the *in vitro* network models we set $\mathcal{E} = \mathcal{E}_c = 0$ and may take $\psi_c = 0$, so we need to evaluate the integral

$$\int_{\psi(0)}^{\psi(t)} \frac{ds}{|J_c - J|^{\Delta_*} f^*\left(s|J_c-J|^{-\beta_*}\right)} = J_c\Lambda_{\max}\frac{t}{\tau}.$$

In Appendix C we show that the scaling function in the denominator may be written $f^*(z) = cv_1(z) + \varphi_*(z)$, where we have estimated both $v_1(z)$ and $\varphi_{1*}(z)$ to order z^5. The two functions are characteristics of the directed percolation universality class, where $\varphi_{1*}(z)$ is a dimensionless counterpart of the effective firing rate nonlinearity $\Phi(\psi)$ and $v_1(z)$ characterizes deviations from the critical point. The constant c weights the deviation $v_1(z)$ (really a "relevant eigenmode" of the RG fixed point) against $\varphi_{1*}(z)$. The sign of this weight depends on the sign of $J_c - J$. For $c < 0$ we expect that $f^*(z)$ has no zeros for all $z > 0$ and the integral will exist for all $0 \leq \psi(t) \leq \psi(0)$. For $c > 0$ $f^*(z)$ may vanish, such that the integral will only exist for s greater than the largest root of $f^*(z)$. We cannot predict the precise value of c with the approximation scheme we use in this work, so we will choose a sufficiently large magnitude for which $f^*(z)$ switches from having no zeros when $c < 0$ to at least one zero when $c > 0$.

We will first derive the general scaling form (5). We first make the change of variables $s' = s|J_c - J|^{-\beta_*}$ and move the factors of $|J_c - J|$ to the right-hand side, giving

$$\int_{y_0}^{y} \frac{ds'}{f^*(s')} = x,$$

where $y = \psi(t)|J_c - J|^{-\beta_*}$, $y_0 = \psi(0)|J_c-J|^{-\beta_*}$, and $x = \Lambda_{\max}|J_c - J|^{\Delta_*-\beta_*}\frac{t}{\tau} = \Lambda_{\max}|J_c-J|^{\nu_* z_*}\frac{t}{\tau}$, where we use our RG scheme's predictions that $z_* = 2$, $\beta_* = \frac{\nu_* d}{2}$, $\Delta_* = \frac{\nu_*}{2}(d+4)$ to reduce $\Delta_* - \beta_* = \nu_* z_*$. Because we are interested in the asymptotic limit of $J \simeq J_c$, we will take y_0 to be large enough that we can replace the lower limit of the integral by $+\infty$. Within this approximation, the left-hand-side is a function of y and the right-hand-side is x, so assuming the function can be inverted we obtain the scaling form (5).

We can derive the asymptotic tails explicitly using the

	$v_1(z)$	$\varphi_1^*(z)$
z	0.22	0.31
z^2	0.067	−0.65
z^3	−0.033	−0.25
z^4	0.025	0.089
z^5	−0.0033	−0.033

TABLE II. Coefficients of the $\mathcal{O}(z^5)$ estimates of the absorbing state network's critical dimensionless nonlinearity $\varphi_1^*(z)$ and the relevant eigenmode $v_1(z)$ in $d = 3$. i.e., the nth row gives the value of $v_1^{(n)}(0)$ or $(\varphi_1^*)^{(n)}(0)$.

results of our renormalization group analysis, covered in Appendix C. We expand $f^*(z) \approx (f^*)'(z_0)(z - z_0) + \frac{1}{2}(f^*)''(z_0)(z - z_0)^2$, cutting the series off at quadratic order, where z_0 is the largest zero of $f^*(z)$. Assuming $y \geq z_0$ we can evaluate the integral analytically, just as in the mean-field case. We obtain the general form $y \sim F(x)$ with

$$F(x) = z_0 + \frac{2(f^*)'(z_0)}{(f^*)''(z_0)} \frac{e^{(f^*)'(z_0)x}}{1 - e^{(f^*)'(z_0)x}}. \quad \text{(A1)}$$

We expect $(f^*)'(z_0) < 0$ and $(f^*)''(z_0) < 0$, such that for large x the scaling variable y approaches z_0 exponentially from above. For $c < 0$ and sufficiently large in magnitude we expect $z_0 = 0$, while for $c > 0$ and large enough we expect $z_0 > 0$.

We can approximate the constants in Eq. (A1) using our $\mathcal{O}(z^5)$ approximation in $d = 3$. If we normalize the eigenmode $v_1(z)$ such that $\int_0^\infty dz\ e^{-z} v_1(z)^2 = 1$, then $|c| = 1.5$ provides a sufficient dividing line between subcritical and supercritical behavior; the actual value could be larger. We find that for $c = -1.5$, $z_0 = 0$ as expected, and $(f^*)'(z_0) = -0.017$ and $(f^*)''(z_0) = -0.75$. For $c = +1.5$ the largest real root occurs at $z_0 = 1.80$ and $(f^*)'(z_0) = -0.74$ and $(f^*)''(z_0) = -0.93$. The coefficients of $v_1(z)$ and $\varphi_1^*(z)$ are given in Table II.

Finally, because $\nu(t) = \Phi_1(\psi(t)) = \Lambda_{\max}^{-1}\psi(t) + |J_c - J|^{\Delta_*} f^*(\psi(t)|J_c - J|^{-\beta_*}) + \ldots$, to leading order $\nu(t)|J_c - J|^{-\beta_*}$ obeys the same scaling form as $y = \psi(t)|J_c - J|^{-\beta_*}$, at least for J close enough to J_c.

in vivo *networks*

For *in vivo* networks we focus on the limit $J \to J_c$, for which $|J_c - J|_*^\Delta f^*((\psi - \psi_c)/|J_c - J|_*^\beta) \to \mathcal{A}[\psi - \psi_c]^{\Delta_*/\beta_*}$, where $\mathcal{A}$ is a universal constant that depends on the spectral dimension d and we define the notation $[\psi - \psi_c]^{\Delta_*/\beta_*} = \text{sgn}(\psi - \psi_c)|\psi - \psi_c|^{\Delta_*/\beta_*}$. We thus need to evaluate the integral

$$\int_{\psi(0)}^{\psi(t)} \frac{ds}{\mathcal{E} - \mathcal{E}_c - \Lambda_{\max}\mathcal{A}[s - \theta^*]^{\Delta_*/\beta_*}} = \frac{t}{\tau}.$$

Because we want to obtain the scaling function corresponding to the scaling form Eq. (6), we make the change of variables $s' = (s - \psi_c)(t/\tau)^{\frac{\beta_*}{\nu_* z_*}}$, giving

$$\int_{y_0}^{y} \frac{ds'}{x - \Lambda_{\max}\mathcal{A}[s']^{\Delta_*/\beta_*}} = 1,$$

where $y = (\psi(t) - \psi_c)(t/\tau)^{\frac{\beta_*}{\nu_* z_*}}$, $y_0 = (\psi(0) - \psi_c)(t/\tau)^{\frac{\beta_*}{\nu_* z_*}}$ and $x = (\mathcal{E} - \mathcal{E}_c)(t/\tau)^{\frac{\Delta_*}{\nu_* z_*}}$, and we used the fact that within our RG scheme we have the approximate scaling relations $(\Delta_* - \beta_*)/\nu_*/z_* = (d + 2 - (d - 2))/2 = 1$. Because we are interested in the asymptotic scaling in the long-time limit $t \gg \tau$, we can take the lower limit y_0 to be $\pm\infty$, depending on whether $\psi(0)$ is above or below θ^*. In addition to the sign of y_0, we also have to consider the sign of x and the sign of y to evaluate the integral.

If $x > 0$ and $y_0 > 0$, we expect that $y > 0$ for all time, and we need to evaluate the integral

$$-\int_{y}^{+\infty} \frac{ds}{x - \Lambda_{\max}\mathcal{A}s^{\Delta_*/\beta_*}} = 1,$$

which requires $x < \Lambda_{\max}\mathcal{A}y^{\Delta_*/\beta_*}$. Similarly, if $x < 0$ and $y_0 < 0$, we expect $y < 0$ for all time and we need to evaluate

$$\int_{-\infty}^{y} \frac{ds}{-|x| + \Lambda_{\max}\mathcal{A}(-s)^{\Delta_*/\beta_*}} = 1,$$

which requires $-|x| + \Lambda_{\max}\mathcal{A}(-y)^{\Delta_*/\beta_*} > 0$. A change of variables $s = -y$ transforms this integral to the $x > 0,\ y > 0$ case.

The next two cases are slightly more complicated. When $x > 0$ but $y_0 < 0$, then we expect y to cross from initially negative values to positive values. For $y < 0$ we may solve

$$\int_{-\infty}^{y} \frac{ds}{x + \Lambda_{\max}\mathcal{A}(-s)^{\Delta_*/\beta_*}} = 1,$$

while for $y > 0$ we need to split up the integral,

$$\int_{-\infty}^{0} \frac{ds}{x + \Lambda_{\max}\mathcal{A}(-s)^{\Delta_*/\beta_*}} + \int_{0}^{y} \frac{ds}{x - \Lambda_{\max}\mathcal{A}s^{\Delta_*/\beta_*}} = 1.$$

Finally, for $x < 0$ but $y_0 > 0$ we expect y to be initially positive and cross over to negative values, so we may write

$$-\int_{y}^{+\infty} \frac{ds}{-|x| - \Lambda_{\max}\mathcal{A}s^{\Delta_*/\beta_*}} = 1,$$

for $y > 0$ and

$$1 = -\int_0^{+\infty} \frac{ds}{-|x| - \Lambda_{\max}\mathcal{A}s^{\Delta_*/\beta_*}} - \int_y^0 \frac{ds}{-|x| + \Lambda_{\max}\mathcal{A}(-s)^{\Delta_*/\beta_*}}.$$

for $y < 0$. For $d = 3$ and $d = 4$ (mean-field) our RG scheme predicts integer values of $\Delta_*/\beta_* = (d+2)/(d-2)$, and the integrals can be evaluated analytically using Mathematica, though the result involves complex-valued representations that ultimately work out to be real and are otherwise not enlightening. The implicit representation cannot be solved in closed form, but can be evaluated numerically to obtain scaling forms for y_+ and y_-, corresponding to the cases $y_0 > 0$ and $y_0 < 0$, respectively. Subtracting these numerical solutions yields the scaling form for $y_+ - y_-$ plotted in Figs. 4D,H,L.

To estimate the asymptotic tails of the distribution for large $x > 0$, we make an additional change of variables $q' = \left(\frac{\Lambda_{\max}\mathcal{A}}{x}\right)^{\frac{\beta_*}{\Delta_*}} s'$. For the $y_0 > 0$ case this gives

$$\begin{aligned}
&\frac{1}{x}\left(\frac{\Lambda_{\max}\mathcal{A}}{x}\right)^{-\frac{\beta_*}{\Delta_*}} \int_q^\infty \frac{dq'}{(q')^{\Delta_*/\beta_*} - 1} \\
&= \frac{1}{x}\left(\frac{\Lambda_{\max}\mathcal{A}}{x}\right)^{-\frac{\beta_*}{\Delta_*}} \int_q^\infty \frac{dq'}{(q'-1)\frac{(q')^{\Delta_*/\beta_*}-1}{q'-1}} \\
&= \frac{1}{x}\left(\frac{\Lambda_{\max}\mathcal{A}}{x}\right)^{-\frac{\beta_*}{\Delta_*}} \left(\left. \frac{\ln(q'-1)}{\frac{(q')^{\Delta_*/\beta_*}-1}{q'-1}} \right|_q^\infty \right. \\
&\qquad \left. - \int_q^\infty dq' \, \ln(q'-1) \frac{d}{dq'}\left(\frac{(q')^{\Delta_*/\beta_*}-1}{q'-1}\right) \right) \\
&\approx -\frac{1}{x}\left(\frac{\Lambda_{\max}\mathcal{A}}{x}\right)^{-\frac{\beta_*}{\Delta_*}} \frac{\ln(q-1)}{\Delta_*/\beta_*} + \ldots
\end{aligned}$$

where in the last line we retain only the leading order behavior as $q \to 1$ and neglect the higher order terms from the integration by parts. Solving for q and writing the result in terms of y_+ gives

$$y_+ \sim 1 + \exp\left(-\frac{\Delta_*}{\beta_*}\left(\frac{\Lambda_{\max}\mathcal{A}}{x}\right)^{\frac{\beta_*}{\Delta_*}} x\right).$$

In the $y_0 < 0$ and $x > 0$ case we make a similar change of variables, and must evaluate

$$\frac{1}{x}\left(\frac{\Lambda_{\max}\mathcal{A}}{x}\right)^{-\frac{\beta_*}{\Delta_*}} \times \left(\int_{-\infty}^0 \frac{dq'}{1-(-q')^{\Delta_*/\beta_*}} + \int_-^q \frac{dq'}{1-(q')^{\Delta_*/\beta_*}}\right).$$

The first integral evaluates to a constant, $\frac{\pi}{\Delta_*/\beta_*}\csc\left(\frac{\pi}{\Delta_*/\beta_*}\right)$, while the second can be evaluated using a similar integration-by-parts trick. The result in terms of y_- is

$$y_- \sim 1 - \exp\left(\pi\csc\left(\frac{\pi}{\Delta_*/\beta_*}\right) - \frac{\Delta_*}{\beta_*}\left(\frac{\Lambda_{\max}\mathcal{A}}{x}\right)^{\frac{\beta_*}{\Delta_*}} x\right).$$

Subtracting $y_+ - y_-$ and omitting constant factors gives a scaling form for the difference of membrane potentials. The $x < 0$ case may be obtained by replacing x with $|x|$. To obtain Eq. (17), which is a scaling form for the spike count differences, we use the fact that $\Phi(\psi(t)) = \nu_c + (J\Lambda_{\max})^{-1}(\psi(t) - \psi_c) + \ldots$, so the leading order scaling is $\nu_+(t) - \nu_-(t) = (J_c\Lambda_{\max})^{-1}(\psi_+(t) - \psi_-(t)) + \ldots$, giving Eq. (17).

Appendix B: Reduction of an excitatory-inhibitory network to a random regular network with effective all-to-all inhibition

The random regular network with all-to-all inhibitory connections considered in Sec. III B is a reduction of a network model with explicit excitatory and inhibitory populations (an "EI" network). In this network the E-E connections follow the random-regular network, while connections to and from the inhibitory population are all-to-all. Here we present the heuristic derivation of this reduction, which is confirmed by the simulation results given in Fig. 4.

We follow the idea of Ref. [84], which performs a similar reduction of a mean-field model for an EI network. The idea behind the derivation is that we want the membrane timescale of the inhibitory population to be fast, such that the membrane potential of the inhibitory neurons very closely follows its input. Second, we introduce synaptic gate dynamics for the inhibitory neurons with synaptic timescale long enough that the filtered spike trains approximately average the rate, allowing us to use a mean-field approximation for the inhibitory spikes.

The dynamics of the inhibitory neurons is given by

$$\begin{aligned}
\tau_I \frac{dV_i(t)}{dt} &= -V_i + \mathcal{E}_I + \sum_{j\in E} J_{ij}\dot{n}_j(t) + \sum_{j\in I} J_{ij}s_j(t), \\
\tau_I^{\text{syn}} \frac{ds_i}{dt} &= -s_i(t) + \dot{n}_i(t), \\
\dot{n}_i(t)dt &\sim \text{Poiss}\left[\phi_I(V_i(t))dt\right], \\
\phi_I(V) &= \gamma\lfloor V_i - \theta_I\rfloor_+,
\end{aligned}$$

where τ_I is the membrane time constant, $\mathcal{E}_I$ is the tonic input to each inhibitory cell, $\sum_{j\in E} J_{ij}\dot{n}_j(t)$ is the input from excitatory neurons, and $\sum_{j\in I} J_{ij}s_j(t)$ is the input from other inhibitory neurons. The variables $s_i(t)$ are the synaptic gating variables of the inhibitory neurons, which essentially amount to an exponential filtering of the inhibitory spike trains with time constant τ_I^{syn}. The spikes are again conditionally Poisson with rate $\phi_I(V)$, which we take to be rectified linear with a gain γ and

threshold θ_I, regardless of whether the E population is an *in vitro* or *in vivo* network. The choice of rectified linear is so that the rate will be linear if the input is above the set-point θ_I.

For τ_I^{syn} and γ large enough, such that there are a reasonably high rate of spikes and $s_i(t)$ is effectively averaging the spikes over time, so we replace the spikes by the rate $\phi_I(V)$. This step is heuristic: the time-average is not equivalent to a trial average because of the non-equilibrium behavior of the network, but for intermediate τ_I^{syn} we expect the difference to not be too large.

Then, for $\tau_I \to 0$, the membrane potential of the spikes essentially follows the input to the cell:

$$\begin{aligned} V_i(t) &\approx \mathcal{E}_I + \sum_{j\in E} J_{ij}\dot{n}_j(t) + \sum_{j\in I} J_{ij}\phi_I(V_j(t)) \\ &= \mathcal{E}_I + \sum_{j\in E} J_{ij}\dot{n}_j(t) + \gamma\sum_{j\in I} J_{ij}(V_j - \theta_I), \end{aligned}$$

where we have assumed that $\mathcal{E}_I$ keeps $V_i(t)$ above θ_I so that the argument of the nonlinearity is positive.

Next, we assume all-to-all connectivity between the populations, and within the inhibitory population [85]:

$$V_i(t) = \mathcal{E}_I + \frac{J_{IE}}{N_E}\sum_{j\in E}\dot{n}_j(t) + \gamma\frac{J_{II}}{N_I}\sum_{j\in I}(V_j - \theta_I);$$

we can solve for the population mean $\psi_I \equiv N_I^{-1}\sum_{i\in i} V_i(t)$ by summing over $i \in I$, giving

$$\psi_I = \frac{\mathcal{E}_I - \gamma J_{II}\theta_I}{1-\gamma J_{II}} + \frac{J_{IE}}{N_E(1-\gamma J_{II})}\sum_{j\in E}\dot{n}_j(t).$$

If the excitatory neurons have the same type of synaptic input $\sum_{j\in I} J_{ij}s_j(t) \approx \frac{J_{EI}}{N_I}\sum_{j\in I}\gamma(V_j(t) - \theta_I)$, then the excitatory dynamics becomes

$$\begin{aligned} \tau\frac{dV_i(t)}{dt} &\approx -V_i + \left(\mathcal{E} - \gamma J_{EI}\theta_I + \gamma J_{EI}\frac{\mathcal{E}_I - \gamma J_{II}\theta_I}{1-\gamma J_{II}}\right) \\ &\quad + \sum_{j\in E}\left(J_{ij} + \frac{1}{N_E}\frac{\gamma J_{EI}J_{EI}}{1-\gamma J_{II}}\right)\dot{n}_j(t) \qquad \text{(B1)} \end{aligned}$$

This is the random regular network with all-to-all inhibitory connections that we consider in Sec. III B. We may set, $\gamma J_{II} = -1$, $\mathcal{E}_I = -\gamma J_{II}\theta_I = +\theta_I$, and $\gamma J_{EI}J_{IE}/(1-\gamma J_{II}) = \gamma J_{EI}J_{IE}/2 = -J(k-2\sqrt{k-1})$ to obtain exactly the model considered. In the simulations shown in Fig. 4I-L, we choose $J_{IE} = J(k-2\sqrt{k-1})$ and $J_{EI} = J_{II} = -0.05J_{IE}$, with $\tau_I = 0.1$ and $\tau_I^{\text{syn}} = 2$, and $\theta_I = -1.0$. We chose the inhibitory connections to not be equal to the excitatory connections so that the excitatory population drives activity in the inhibitory population.

Appendix C: Renormalization group analysis of the stochastic spiking network model

In this section we give an overview of the renormalization group method we developed to analyze the spiking network model and obtain the renormalized scaling theory. We will first give a conceptual overview of the key ideas of the renormalization group, before describing technical details of the method.

1. Renormalization in concept

Conceptually, the renormalization group (RG) method describes how our mathematical descriptions of physical processes change when we view them at a hierarchy of coarser and coarser scales. The prototypical example of a hierarchy of scales in physics is length scale: as we "zoom out" from the microscopic scale, processes or structures with fine detail become less resolved, blurring into an overall larger picture. Depending on the state of the system, as we zoom out a system with a mix of microscopic disordered and ordered regions may look increasingly ordered or increasingly disordered on coarser scales. At a critical point it may be hard to tell the difference: at each zoom level the system looks statistically similar, owing to the (incomplete) scale invariance caused by processes of all orders being strongly coupled.

A challenge for applying the RG to neural circuitry is that is it not clear what the appropriate hierarchy of scales is. Coarse graining over spatial scales may not be informative, as connections between neurons may be long range and so such a coarse graining may erroneously blur together neurons whose activities are not strongly coupled. Data driven approaches, like the phenomenological renormalization group developed by Ref. [22], have proposed using the eigenvalues of the spike-spike correlations—the "principal components—as the coarse-graining space, and have shown that the method appears to yield good scaling collapses of hippocampal data. However, the difficulty with using principal components in a theoretical analysis is that it requires we solve the model first so that we can compute the principal components. Instead, in this work we show that one fruitful choice of hierarchy of scales is the eigenvalue distribution of the synaptic weight matrix J_{ij}. If the synaptic connections between neurons are organized in a lattice, then this choice effectively reduces to coarse-graining over "energy shells," which is related to the classic momentum-shell renormalization scheme common in physics. We illustrate the effect of coarse-graining the eigenspace of the synaptic connections in Fig. 6. In the RG scheme we use in this work, which is based on the "non-perturbative renormalization group" (NPRG) method, eigenmodes up to a given eigenvalue threshold Λ are progressively incorporated into estimates of the network statistics. When $\Lambda < \Lambda_{\text{min}}$, the smallest eigenvalue of the synaptic weight matrix, the network is decouples into independent Pois-

son neurons, whose statistics can be evaluated exactly. As $\Lambda \to \Lambda_{\max}$, all modes are incorporated. The RG method provides a means of relating the network statistics at a particular value of Λ to the statistics at $\Lambda - d\Lambda$, providing an interpolation from the independent network to the network of interest.

Developing a renormalization group scheme to study spiking network models is non-trivial, and to date has not been done for the leaky integrate-and-fire model considered here, only for network models of units with "active" and "quiescent" states that can be loosely interpreted as "spiking" and "non-spiking" [12, 16], or for networks characterized by coarse-grained firing rates, rather than spiking activity [33, 34].

The RG method we use in this work is based on the non-perturbative renormalization group (NPRG) method, which has been successfully used to study many problems in condensed matter physics [75, 86–96]. For pedagogical introductions, see [97] for equilibrium systems, [86, 89, 98, 99] for non-equilibrium systems, and [100] for a broad overview. However, because these methods have been developed for lattices or continuous media in which the fluctuations are driven by Gaussian noise, they cannot be straightforwardly applied to spiking network models.

In order to implement the RG scheme in practice, we will formulate the stochastic system of equations for the network as a statistical field theory, to which the methods of the non-perturbative renormalization group can be adapted and applied.

2. Renormalization in practice

a. Field theoretic formulation of the spiking network

We convert Eqs. (1)-(2) into a field theory using the Martin-Siggia-Rose-Janssen-De Dominicis (MSRJD) path integral formalism for stochastic differential equations [101–104]. The probability of the joint membrane and spiking dynamics can be represented as a functional integral $P[V, \dot{n}] = \int \mathcal{D}[\tilde{V}, \tilde{n}] \; \exp(-S[\tilde{V}, V, \tilde{n}, \dot{n}])$, defining the action [103, 104]

$$S[\tilde{V}, V, \tilde{n}, \dot{n}] = \sum_{i=1}^{N} \int_{-\infty}^{\infty} dt \; \left\{ \tilde{V}_i(t) \left(\tau \frac{dV_i(t)}{dt} + V_i(t) - \mathcal{E} - \sum_{j=1}^{N} J_{ij} \dot{n}_j(t) \right) + \tilde{n}_i(t)\dot{n}_i(t) - \left(e^{\tilde{n}_i(t)} - 1 \right) \phi\left(V_i(t)\right) \right\}; \tag{C1}$$

$\tilde{V}$ and $\tilde{n}$ and the auxiliary "response fields" that arise in the construction of the path integral. The term $\left(e^{\tilde{n}_i(t)} - 1\right) \phi\left(V_i(t)\right)$ arises from choosing the conditional spike probabilities to be Poisson or Bernoulli. We do not explicitly write the terms corresponding to initial conditions, as these can be implemented through the source terms to be introduced. To lighten notation going forward, we will use the shorthands $a \cdot b = \sum_{i,\alpha} \int dt \; a_i^\alpha(t) b_i^\beta(t)$ and $a \cdot M \cdot b = \sum_{ij,\alpha\beta} \int dt dt' \; a_i^\alpha(t) M_{ij}^{\alpha\beta}(t-t') b_j^\beta(t')$, where i, j run over neuron indices, α, β index the different fields $\{\tilde{V}, V, \tilde{n}, \dot{n}\}$ (or their corresponding sources, to be introduced), and $t, t' \in \mathbb{R}$ are times.

The mean-field theory of the model, Eqs. (7), formally corresponds to a saddle-point approximation of the probability $P[V, \dot{n}]$, taking variational derivatives of the action with respect to each of the four types of fields, with the steady-state condition imposing $\tilde{V} = \tilde{n} = 0$. This field theory was first developed for the spiking dynamics (marginalized over $V, \tilde{V}$) by [101], who also developed diagrammatic rules for calculating the perturbative corrections to the mean-field approximation. This perturbative formalism is useful for improving predictions of network statistics in parameter regimes far from phase transitions, but accurately predicting statistics in the vicinity of a transition requires a renormalization group approach to extend the validity of perturbative approaches to the critical point. Typical perturbative RG treatments in statistical physics rely on the interactions between units being translation invariant, such that the field theory can be Fourier transformed into momentum-space, allowing for integrating out modes within narrow momentum bands, facilitating perturbative calculation of the RG flow equations. While many models of neural activity have been formulated using lattices or translation-invariant connections, these are seldom realistic models of neural wiring, and it would be desirable to have an RG formalism that does not rely on translation invariance. For this purpose we turn to the NPRG.

b. The non-perturbative renormalization group extended to the spiking network model

The key mathematical idea behind the NPRG method is to define a one-parameter family of models that interpolates from a solvable limit of the model to the full theory by means of a differential equation that is amenable to tractable variational approximations, rather than relying on perturbative approximations.

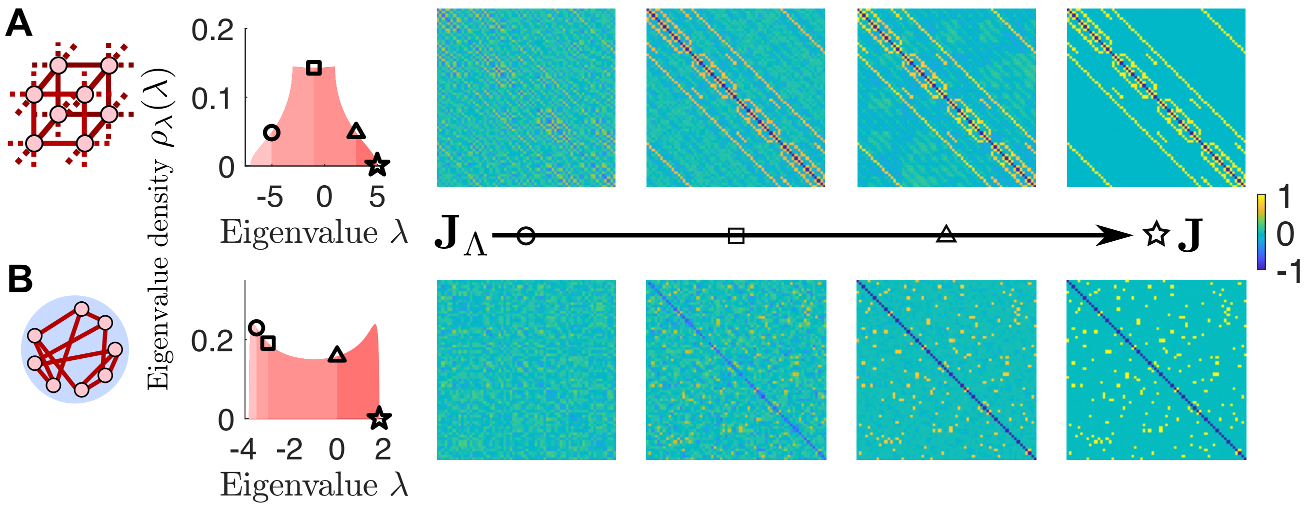

FIG. 6. **Regulation of the synaptic weight matrix by thresholding eigenvalues**. Our renormalization group flow is implemented by thresholding the eigenvalues of the synaptic weight matrices, e.g., for **A)** a 3-dimensional lattice, and **B)** random regular networks in which the number of connections each neuron makes is fixed but the pairs of neurons connected are otherwise random. The thresholding procedure sets all eigenvalues greater than a threshold Λ equal to zero. The full network is recovered as $\Lambda \to \Lambda_{\max}$. Note that, as in the case of the random regular network, it is possible that some eigenvalues are isolated away from the continuous spectrum. These eigenvalues, however, can be moved by appropriate modifications of the network connections; see Sec. III B.

The variation of the NPRG we adapt starts by modifying the moment generating functional (MGF) $\mathcal{Z}[\mathcal{A}]$ or the related cumulant generating functional (CGF) $\mathcal{W}[\mathcal{A}] = \ln \mathcal{Z}[\mathcal{A}]$,

$$\begin{aligned}\mathcal{Z}[\mathcal{A}] &\equiv \exp\left(\mathcal{W}[\mathcal{A}]\right) \qquad\qquad (C2)\\ &= \int \mathcal{D}[\tilde{V}, V, \tilde{n}, \dot{n}]\; e^{-S[\tilde{V},V,\tilde{n},\dot{n}]+\tilde{V}\cdot h+V\cdot\tilde{h}+\tilde{n}\cdot j+\dot{n}\cdot\tilde{j}}.\end{aligned}$$

The MGF and CGF are functionals of "source fields" $\mathcal{A} = \{h, \tilde{h}, j, \tilde{j}\}$. Note that we use the convention of pairing fields with tildes to their partners without tildes, as all fields with tildes may be taken to be purely imaginary. Derivatives of the MGF evaluated at zero sources would yield statistical moments and response functions of the joint spike train and membrane potential statistics, while derivatives of the CGF yield cumulants or centered moments and response functions.

Computing $\mathcal{Z}[\mathcal{A}]$ or $\mathcal{W}[\mathcal{A}]$ exactly would therefore constitute an exact solution of the stochastic spiking model. In practice, this is intractable, except in special cases. One such special case is the limit of no synaptic coupling: $J_{ij} = 0$. In this case the neural spikes are just independent Poisson processes driven by membrane potentials that sit at the baseline $\mathcal{E}$. This motivates the choice of regulating the synaptic weights between neurons by replacing J_{ij} with $J_{ij;\Lambda}$, where Λ parametrizes the family of models that interpolates from the network of independent neurons to the actual network we want to study.

Because we need only interpolate between these two endpoints, there are many choices we could make for Λ. For the symmetric networks we consider in this work a natural choice is to use Λ as a threshold on the eigenvalues of the synaptic weight matrix, defining $J_{ij;\Lambda}$ by setting to 0 any eigenvalues greater than Λ. This choice defines a family of MGFs, $\mathcal{Z}_\Lambda[\mathcal{A}]$, indexed by the value of the eigenvalue threshold $\Lambda \in [\Lambda_{\min}, \Lambda_{\max}]$, where $\Lambda_{\min}$ and $\Lambda_{\max}$ are the smallest and largest eigenvalues of the synaptic weight matrix. We can relate the MGF at one value of Λ to its value at $\Lambda + d\Lambda$ by taking a partial derivative of the definition (C2). The derivative will bring down a factor of $\sum_{ij} \int dt dt'\; \tilde{V}_j(t') \partial_\Lambda J_{\Lambda;ij}(t-t') \dot{n}_j(t')$ inside the path integral; the factors of $\tilde{V}_i(t)$ and $\dot{n}_j(t')$ can be replaced with variational derivatives with respect to their conjugate sources, allowing us to pull the differential operator outside of the path integral, giving

$$\partial_\Lambda \mathcal{Z}_\Lambda[\mathcal{A}] = \sum_{ij} \int dt dt'\; \partial_\Lambda J_{\Lambda;ij} \frac{\delta^2 \mathcal{Z}_\Lambda[\mathcal{A}]}{\delta h_i(t) \delta \tilde{j}_j(t')}. \qquad (C3)$$

While this looks like a kind of linear differential equation for the functional $\mathcal{Z}_\Lambda[\mathcal{A}]$, in practice it is actually more useful to transform this into a flow equation for a related object, the *average effective action* (AEA) $\Gamma[\chi]$. The regulated AEA is defined as a modified Legendre transform of the CGF $\mathcal{W}_\Lambda[\mathcal{A}]$,

$$\Gamma_\Lambda[\chi] = -\mathcal{W}_\Lambda[\mathcal{A}] + \chi \cdot \mathcal{A} - \frac{1}{2} \chi \cdot [J - J_\Lambda] \cdot \chi, \qquad (C4)$$

where $\chi = \{\tilde{\psi}, \psi, \tilde{\nu}, \nu\}$ can be thought of as the expected values of the fields $\{\tilde{V}, V, \tilde{n}, \dot{n}\}$, respectively, in the presence of the sources. The conjugate sets of fields are de-

fined as functions of each other via the relations

$$\chi_i(t) = \frac{\delta \mathcal{W}[\mathcal{A}]}{\delta \mathcal{A}_i(t)}, \text{ or } \mathcal{A}_i(t) = \frac{\delta \Gamma[\chi]}{\delta \chi_i(t)}, \tag{C5}$$

allowing conversion between the CGF and the AEA. The term $J - J_\Lambda$ in Eq. (C4) couples only $\tilde{\psi}$ and ν fields. By construction, $\Gamma_{\Lambda=\Lambda_{\min}} = S[\chi]$ is the mean-field theory of the spiking network model and $\Gamma_{\Lambda=\Lambda_{\max}}[\chi] = \Gamma[\chi]$, the true AEA of the model. Note that while the mean-field equations (7) derive from the saddle-points of the action S, the equations of motion for the *true* means are saddle-points of the AEA Γ.

Adapting the derivation in Ref. [99], we can show the AEA obeys the celebrated Wetterich flow equation [105],

$$\partial_\Lambda \Gamma_\Lambda = \frac{1}{2} \text{Tr} \left[\partial_\Lambda \mathbf{R}_\Lambda \cdot \left[\Gamma^{(2)}_\Lambda + \mathbf{R}_\Lambda \right]^{-1} \right], \tag{C6}$$

where Tr denotes a super-trace over field indices χ, neuron indices, and times. $\Gamma^{(2)}_\Lambda$ is a $4N \times 4N$ matrix of second derivatives of Γ_Λ with respect to pairs of the fields χ, $\mathbf{R}_\Lambda = \mathbf{J} - \mathbf{J}_\Lambda$, and the factor $\left[\Gamma^{(2)}_\Lambda + \mathbf{R}_\Lambda\right]^{-1}$ is an inverse taken over matrix indices, field indices, and time.

The Wetterich equation is exact, but being a functional integro-partial differential equation it cannot be solved in practice, and approximations are still necessary. Despite the flow equation for (C6) appearing much more complicated than (C3), the advantage of using $\Gamma_\Lambda[\chi]$ over $\mathcal{Z}_\Lambda[\mathcal{A}]$ is that the AEA shares much of its structure with the original action S, allowing us to better constrain our non-perturbative approximation. The standard approach is to make an *ansatz* for the form of the solution, constrained by symmetries or Ward-Takahashi identities, and employing physical intuition. The action of the spiking network model does not readily admit any obvious symmetries, but we can derive a pair of Ward-Takahashi identities that allows us to restrict the form of the AEA.

The common approach to deriving WT identities is to perturb a field by an infinitesimal amount and demand the resulting linear variation in the action vanishes [94]. However, an alternative approach is available for the spiking model. In the spiking model we can analytically integrate out either the membrane potential fields or the spiking fields when evaluating the MGF, leaving a path integral over the remaining pair of fields to be performed. If we integrate out the membrane potential fields and then differentiate the MGF with respect to $\tilde{h}_i(t)$, we obtain the identity

$$\left(\tau \frac{d}{dt} + 1\right) \frac{\delta \mathcal{Z}[\mathcal{A}]}{\delta \tilde{h}_i(t)} = (h_i(t) + \mathcal{E}) \mathcal{Z}[\mathcal{A}] + \sum_j J_{ij} \frac{\delta \mathcal{Z}[\mathcal{A}]}{\delta \tilde{j}_j(t)}.$$

If we integrate out the spike fields and differentiate with respect to the source $\tilde{j}_i(t)$, we obtain the identity

$$\frac{\delta \mathcal{Z}[\mathcal{A}]}{\delta j_i(t)} = \tilde{j}_i(t) Z[\mathcal{A}] + \sum_j \frac{\delta \mathcal{Z}[\mathcal{A}]}{\delta h_j(t)} J_{ji}.$$

Using the definition $\mathcal{Z}_\Lambda = \exp(\mathcal{W}_\Lambda)$ to write these identities in terms of the CGF $\mathcal{W}_\Lambda$ and then using the relationships (C5) to replace sources with variational derivatives of Γ and derivatives of $\mathcal{W}$ with the expectation fields, we conclude that the AEA must have the form

$$\Gamma[\tilde{\psi}, \psi, \tilde{\nu}, \nu] = \sum_{i=1}^N \int_{-\infty}^{\infty} dt \, \left\{ \tilde{\psi}_i(t) \left(\tau \frac{d\psi_i(t)}{dt} + \psi_i(t) - \mathcal{E}_i - \sum_{j=1}^N J_{ij} \nu_j(t) \right) + \tilde{\nu}_i(t) \nu_i(t) \right\} + \Upsilon[\tilde{\nu}, \psi], \tag{C7}$$

where J is the true synaptic coupling, not the regulated coupling J_Λ, and the functional $\Upsilon[\tilde{\nu}, \psi]$ couples only the spike-response fields $\tilde{\nu}$ and the membrane-potential fields ψ.

Our result for Eq. (C7) shows that the membrane dynamics are unrenormalized by stochastic fluctuations—only the interactions between the membrane potential and the spiking statistics are renormalized, and we need only derive the RG flow for the functional $\Upsilon_\Lambda[\tilde{\nu}, \psi]$. To do so, we exploit the fact that the networks we consider in this application have a leading homogeneous mode (i.e., $\sum_{j=1}^N J_{ij} = J \Lambda_{\max}$). We follow previous NPRG work by performing a "local potential approximation (LPA)" in which we set the fields to time- and index-independent values $\tilde{\nu}_i(t) = \tilde{\nu}$ and $\psi_i(t) = \psi$. This reduces Υ to a function we need to solve for, not a functional. We define the "local potential" U_Λ by

$$\Upsilon_\Lambda[\tilde{\nu}, \psi] \Big|_{\tilde{\nu}_i(t) = \tilde{\nu}, \; \psi_i(t) = \psi} \equiv -NTU_\Lambda(\tilde{\nu}, \psi), \tag{C8}$$

where square brackets denote functionals and round brackets denote a function. The proportionality factors N and T are the number of neurons and the duration of the spike train, which become infinite but cancel out of the flow equation.

Using this approximation, we compute the functional derivatives of Γ_Λ in the matrix $\Gamma^{(2)}$, *and then* evaluate them at homogeneous values $\tilde{\nu}_i(t) \to \tilde{\nu}$ and $\psi_i(t) \to \psi$. After inserting the homogeneous fields, it is possible to invert $\mathbf{\Gamma}^{(2)}_\Lambda + \mathbf{R}_\Lambda$ in closed form. The super-trace Tr involves a sum over the four fields, neural indices, and temporal frequency. For symmetric matrices $\mathbf{J}$ the matrices

can be diagonalized, and the cyclic nature of the trace causes the eigenvectors to drop out of the flow equation. The result in the $N \to \infty$ limit is

$$\partial_\Lambda U_\Lambda(\tilde{\nu}, \psi) = \frac{1}{2\tau}\rho_\lambda(\Lambda)\left\{1 - \Lambda U^{(1,1)}_\Lambda(\tilde{\nu}, \psi) - \sqrt{\left(1 - \Lambda U^{(1,1)}_\Lambda(\tilde{\nu}, \psi)\right)^2 - \Lambda^2 U^{(0,2)}_\Lambda(\tilde{\nu}, \psi) U^{(2,0)}_\Lambda(\tilde{\nu}, \psi)}\right\}, \tag{C9}$$

where $\rho_\lambda(\lambda)$ is the eigenvalue density of J_{ij} and the initial condition is $U_{\Lambda=\Lambda_{\min}}(\tilde{\nu}, \psi) = (e^{\tilde{\nu}} - 1)\phi(\psi)$. Note that in the remainder of this appendix we absorb the synaptic strength J into the definition of the eigenvalues.

In practice, we do not solve Eq. (C9) directly. Instead, we expand $U_\Lambda(\tilde{\nu}, \psi) = \sum_{m=1}^\infty \frac{\tilde{\nu}^m}{m!}\Phi_{m,\Lambda}(\psi)$, introducing an infinite set of effective nonlinearities $\Phi_{\Lambda,m}(\psi)$. We obtain a hierarchy of partial differential equations for these nonlinearties by differentiating Eq. (C9) m times with with respect to $\tilde{\nu}$ and then setting $\tilde{\nu} = 0$. This procedure will yield the hierarchy of flow equations of the form

$$\partial_\Lambda \Phi_{m,\Lambda}(\psi) = \rho_\lambda(\Lambda)\mathcal{F}_m(\Phi_{1,\Lambda}, \ldots, \Phi_{m,\Lambda}, \Phi_{m+1,\Lambda}). \tag{C10}$$

The functions $\mathcal{F}_m$ depend on the nonlinearities as well as derivatives of those nonlinearities with respect to ψ, which are not denoted explicitly as arguments. The primary nonlinearity of interest will be the nonlinearity $\Phi(\psi) \equiv \Phi_{1,\Lambda_{\max}}(\psi)$, which appears in the renormalized equations for the dynamics of the trial-and-population-averaged means introduced in Sec. II C, Eqs. (12)-(13). However, to understand the origin of anomalous critical exponents at the phase transitions in both the *in vitro* and *in vivo* networks, it will be necessary to compute contributions from higher order nonlinearities.

The hierarchy at level m depends only on the next order nonlinearity $\Phi_{m+1,\Lambda}(\psi)$. This provides a convenient means of closing the hierarchy and solving a finite system of equations for the nonlinearities. We close the hierarchy at order m by making the approximation $\Phi_{m+1,\Lambda}(\psi) \approx \Phi_{1,\Lambda}(\psi)$. This closure approximation is motivated by the idea that closing the hierarchy at $m = 2$ would amount to the approximation $U_\Lambda(\tilde{\nu}, \psi) = (e^{\tilde{\nu}} - 1)\Phi_{1,\Lambda}(\psi)$; i.e., the nonlinearity is renormalized but not the "Poissonness" of the spiking fluctuations. Cutting the hierarchy off at higher orders m amounts to truncating an expansion of U_Λ in powers of $e^{\tilde{\nu}} - 1$, effectively limiting how non-Poisson the spiking becomes.

As a concrete example, the flow equations for $\Phi_{1,\Lambda}(\psi)$ and $\Phi_{2,\Lambda}(\psi)$ work out to be

$$\partial_\Lambda \Phi_{1,\Lambda}(\psi) = \frac{\rho_\lambda(\Lambda)\Lambda^2}{4\tau}\frac{\Phi_{2,\Lambda}(\psi)\Phi''_{1,\Lambda}(\psi)}{1 - \Lambda\Phi'_{1,\Lambda}(\psi)} \tag{C11}$$

$$\partial_\Lambda \Phi_{2,\Lambda}(y) = \frac{\rho_\lambda(\Lambda)\Lambda^2}{8\tau}\left[\frac{\Lambda^2\Phi_{2,\Lambda}(y)^2\Phi''_{1,\Lambda}(y)^2}{(1 - \Lambda\Phi'_{1,\Lambda}(y))^3} + \frac{4\Lambda\Phi_{2,\Lambda}(y)\Phi'_{2,\Lambda}(y)\Phi''_{1,\Lambda}(y)}{(1 - \Lambda\Phi'_{1,\Lambda}(y))^2} + \frac{4\Phi_{3,\Lambda}(y)\Phi''_{1,\Lambda}(y) + 2\Phi_{2,\Lambda}(y)\Phi''_{2,\Lambda}(y)}{1 - \Lambda\Phi'_{1,\Lambda}(y)}\right]. \tag{C12}$$

We remind the reader that in these equations we have absorbed the synaptic strength J into the definition of the eigenvalue Λ. The initial initial conditions are $\Phi_{m,\Lambda_{\min}}(\psi) = \phi(\psi)$, with boundary conditions $\Phi_{m,\Lambda}(\psi) \overset{\lim|\psi|\to\infty}{\sim} \phi(\psi)$ for *in vivo* networks and $\Phi_{m,\Lambda}(0) = 0$ for *in vitro* networks, where we take $\psi = 0$ to be the activation threshold. The numerical solutions for $\Phi(\psi) = \Phi_{1,\Lambda_{\max}}(\psi)$ plotted in Fig. 7 are obtained by numerically solving Eq. (C11) up to fourth order ($m = 3, 4$ equations not shown).

While we cannot solve the hierarchy of equations in closed form, we show next that we can analytically extract the anomalous scaling behavior when the network is tuned close to a critical point. This anomalous behavior emerges in the solutions of the hierarchy (C10) when $1 - \Lambda\Phi'_{1,\Lambda}(\psi)$—the denominator in Eq. (C11), which appears in all equations in the hierarchy—vanishes. This factor is finite when the network is in the subcritical regime and $\Lambda < \Lambda_{\max}$, but at a critical value of $J = J_c$ there is a point $\psi = \psi_c$ at which the denominator vanishes at the end of the flow. The overall solution does not diverge, but becomes non-analytic, depending on non-integer powers of $\psi - \psi_c$. For $J > J_c$ we expect the denominator to remain finite, though possibly small, for $\Lambda < \Lambda_{\max}$, but as $\Lambda \to \Lambda^-_{\max}$ the denominator $1 - \Lambda_{\max}\Phi'_{1,\Lambda}(\psi)$ vanishes for all $\psi \in [\psi_-, \psi_+]$ and

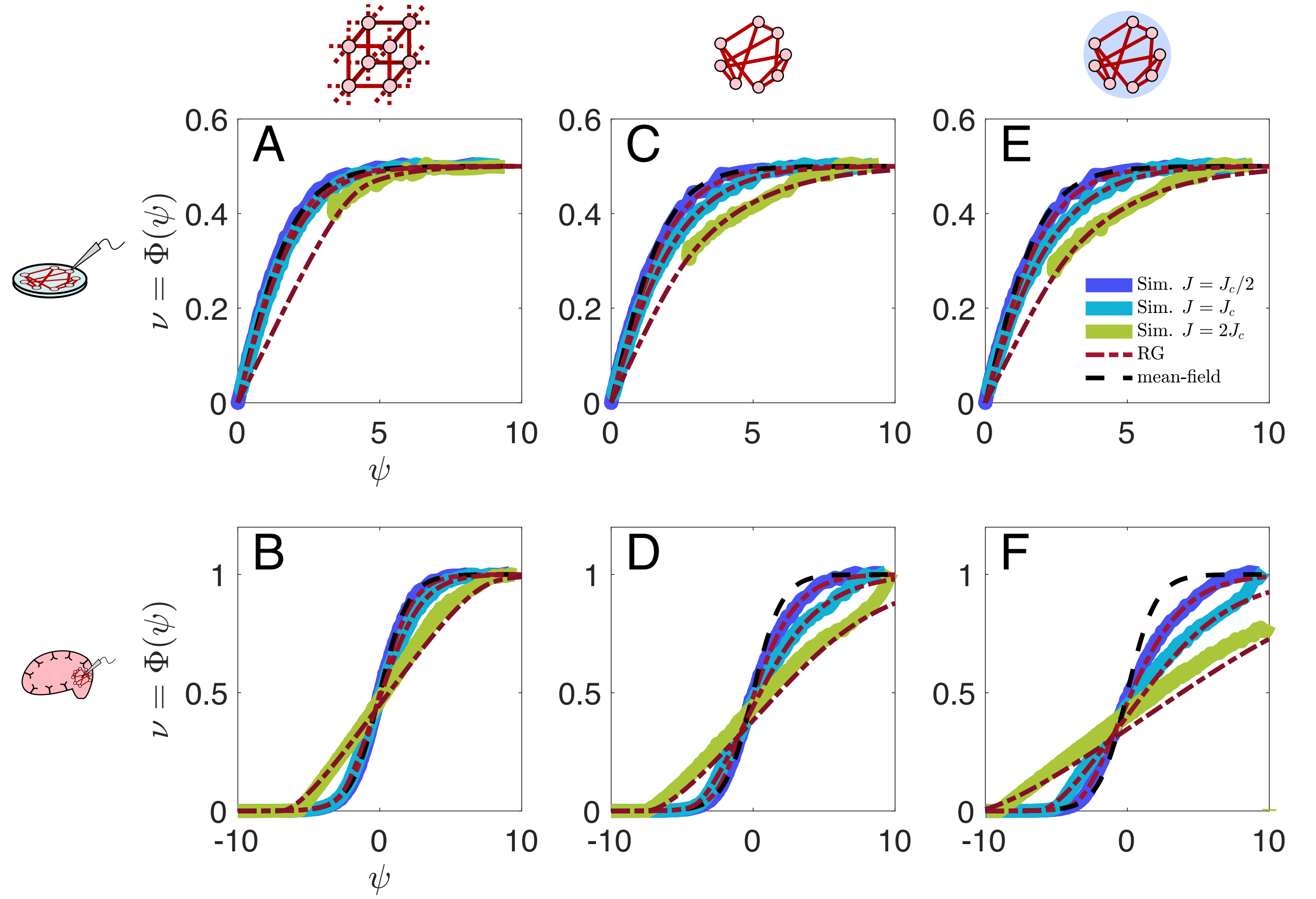

FIG. 7. **Effective nonlinearities on different networks with effective dimension** $d = 3$ and synaptic strengths $J = \{J_c/2, J_c, 2J_c\}$. **A-B)** Effective nonlinearities for a cubic lattice of $N = 14^3$ neurons for an absorbing state network (**A**) with $J_c \approx 0.825$ and a spontaneous network (**B**) with $J_c \approx 1.165$. **C-D)** Effective nonlinearities for an excitatory random regular network of $N = 2^{11}$ neurons for an absorbing state network (**C**) with $J_c \approx 2.17$ and a spontaneous network (**D**) with $J_c \approx 3.75$. **E-F)** Effective nonlinearities for an effective excitatory-inhibitory population with sparse excitatory-excitatory connections and dense (all-to-all) connections between other pairs, with $N = 2^{11}$ neurons, for an absorbing state network (**E**) with $J_c \approx 2.32$ and a spontaneous network (**F**) with $J_c \approx 6.0$. In all cases the blue data points are simulated data averaged over 100 trials. The red curves are the predictions of the hierarchy of nonlinearities (Eqs. (C10)) with the truncation $\Phi_{5,\Lambda}(\psi) \approx \Phi_{1,\Lambda}(\psi)$. The black dashed lines correspond to the mean-field prediction, $\Phi_{1,\Lambda}(\psi) = \phi(\psi)$. The curves in the supercritical phases are less accurate because the solution becomes non-analytic, which is difficult to capture numerically.

$\Phi_{1,\Lambda_{\max}}(\psi)$ becomes exactly linear on this interval, as seen in Fig. 7. The end-points $\psi_\pm$ represent the possible metastable states of the network in the supercritical phase, and the interval $[\psi_-, \psi_+]$ represents a coexistence region between metastable states. This is directly analogous to the development of the non-analyticity in the free-energy of the Ising model in the ordered phase [18].

c. *Universality in the renormalization group flow*

So far, our RG treatment of the spiking network model has implemented the first step of an RG procedure, coarse-graining. To extract the anomalous scaling behavior of the nonlinearities close to the critical point, we need to implement the second RG step, rescaling.

In the NPRG context, the rescaling procedure will amount to identifying an appropriate non-dimensionalization of the hierarchy of flow equations Eq. (C10) and searching for fixed point solutions. We make the change of variables

$$\Phi_{1,\Lambda}(\psi) = \Pi_s + \Lambda_{\max}^{-1}(\psi - \theta_s) + e^{-s(d/2+1-\eta_s^X)}\varphi_{1,s}\left((\psi - \theta_s)e^{s(d/2-\eta_s^X)}\right), \tag{C13}$$

$$\Phi_{m,\Lambda}(\psi) = e^{-s(d/2+1-m\eta_s^X)} \times \varphi_{m,s}\left((\psi - \theta_s)e^{s(d/2-\eta_s^X)}\right), \tag{C14}$$

where we define the "RG-time"

$$s \equiv -\ln\left(\frac{\Lambda_{\max}-\Lambda}{\Lambda_{\max}-\Lambda_{\min}}\right) \in [0,\infty); \tag{C15}$$

we define s to be positive, in contrast to the convention in some NPRG works. In the first nonlinearity we remove the running baseline firing rate $\Pi_s = \Phi_{1,\Lambda}(\theta_s)$ and the critical slope $\Lambda_{\max}^{-1}(\psi-\theta_s)$, as these would become infinite offsets under our rescaling procedure. In the *in vitro* networks $\Pi_s = 0$ and $\theta_s = 0$, as stochastic fluctuations do not generate activity-independent spiking, nor do they shift the activation threshold of the effective nonlinearity.

This change of variables introduces the "dimensionless" nonlinearities $\varphi_{m,s}(z)$, which represent centered and rescaled versions of the nonlinearities $\Phi_{m,\Lambda}(\psi)$, where $z \equiv (\psi - \theta_s)e^{s(d/2-\eta_s^X)}$ is the dimensionless membrane potential centered on the running set-point $\theta_s = \text{argmax}\ \Phi'_{1,\Lambda}(\psi)$. The factor $e^{s(d/2-\eta_s^X)}$ is a "running" scale-factor that zooms in on this point, where d is the spectral dimension of the network (Eq. (15)). Similarly, the factors $e^{-s(d/2+1-m\eta_s^X)}$ scale down the amplitudes of the nonlinearities $\Phi_{m,\Lambda}(\psi)$; the factor $e^{sm\eta_s^X}$ originates from a rescaling of the spike response field $\tilde{\nu} = \tilde{z}e^{-s\eta_s^X}$, which defines the running exponent η_s^X, to be determined shortly. We omit some dimensional constant factors that depend on the eigenvalue density and initial values of the couplings, as these do not contribute to the critical exponents.

Our choice of scaling factors renders the flow equations for $\varphi_{m,s}(z)$ asymptotically independent of s for $s \gg 1$. e.g.,

$$\partial_s\varphi_{1,s}(z) - \left(\frac{d}{2}+1-\eta_s^X\right)\varphi_{1,s}(z) + \left[\left(\frac{d}{2}-\eta_s^X\right)z - \zeta_s\right]\varphi'_{1,s}(z) = \frac{1}{2}\frac{\varphi_{2,s}(z)\varphi''_{1,s}(z)}{1-\varphi'_{1,s}(z)}, \tag{C16}$$

with similar but more complicated equations for higher order $\varphi_{m,s}(z)$. There is also a flow equation for Π_s, but while it is driven by the flow of the $\varphi_{m,s}(z)$, it does not couple back into the nonlinearities. When searching for fixed points of the flow equation we may thus ignore the flow of Π_s, though it will converge to a (non-universal) value ν_c at the end of the flow that determines the mean firing rate at the critical point.

In *in vivo* networks the term $\zeta_s \equiv e^{s(d/2-\eta_s^X)}\partial_s\theta_s$ is necessary to fix $\varphi''_{1,s}(0) = 0$ for all s throughout the flow, which follows from centering our rescaling point around $\theta_s = \text{argmax}\ \Phi'_1(\psi)$. ζ_s depends on several derivatives of $\varphi_{m,s}(z)$ at $z = 0$, and we will find that at the critical point $\zeta_* = 0$. In *in vitro* networks $\theta_s = 0$, and hence $\zeta_s = 0$.

Similarly, η_s^X is chosen to either impose a relationship between derivatives (as we will do for *in vitro* networks) or fix one of the derivatives of the nonlinearites to 1 (as we will do for *in vivo* networks), and will depend on derivatives of $\varphi_{m,s}(z)$ at $z = 0$.

Although Eq. (C16) is only valid for RG-times $s \to \infty$, we retain some autonomous time-dependence for the purposes of performing linear stability analyses around fixed points of the flow. That is, we will expand the dimensionless nonlinearities around their fixed points, $\varphi_{m,s}(z) = \varphi_{m*}(z) + e^{\mu_\ell s}v_{m,\ell}(z)$, to obtain a system of equations for the eigenmodes of the RG flow, $v_{m,\ell}(z)$, and their associated eigenvalues μ_ℓ. If $\mu_\ell > 0$ the eigenmode is "relevant," and the RG flow is repelled away from the critical point along the directions $v_{m,\ell}(z)$. If $\mu_\ell < 0$ the eigenmode is "irrelevant," and projections of the dimensionless nonlinearities onto the modes $v_{m,\ell}(z)$ will decay. However, despite this decay, we will show in Sec. C 2 f that the irrelevant eigenmodes are important for understanding the shape of the effective nonlinearity $\Phi_{1,\Lambda}(\psi)$.

We cannot solve Eq. (C16) and its higher order siblings exactly, as they are nonlinear partial differential equations, so we use a combination of perturbative and non-perturbative techniques to estimate the fixed points. Our scheme involves first estimating the fixed points with a low-order non-perturbative approximation, which gives a qualitative picture of the transition and determines the appropriate expansion parameter to use in a perturbative solution of our flow equations for dimensions close to the upper critical dimension, the dimension above which the only stable fixed point of the flow equation is trivial. This perturbative solution can then be used to seed an iterative root-finding routine to solve for the fixed points of a higher order non-perturbative expansion. Here we only show the minimal truncation to illustrate the qualitative ideas of the truncation procedure, and quote the results of the higher order truncations.

The non-perturbative method involves expanding $\varphi_{m,s}(z)$ in a series around $z = 0$ and truncating at a finite order,

$$\varphi_{m,s}(z) = \sum_{n=1}^{\infty}\frac{g_{mn,s}}{n!}z^n, \tag{C17}$$

where we have defined the dimensionless running couplings $\varphi_{m,s}^{(n)}(0) \equiv g_{mn,s}$. Truncating this series does not reflect an assumption that the variable z is small, but constitutes a further variational projection onto a reduced solution subspace, similar to how RG analyses of

the Ising model often track only the flow of two couplings, despite coarse graining generating couplings of all orders. After choosing a finite number of couplings to consider, we obtain a system of differential equations by differentiating the flow equations for $\varphi_{m,s}(z)$ with respect to the appropriate powers of z and evaluating at $z = 0$.

For a low order truncation in just a few of the $g_{mn,s}$ terms we can analytically solve for the fixed points and determine their dependence on the difference between the upper critical dimension d_c and the spectral dimension d. A linear stability analysis around these fixed points also gives an estimate of the critical exponent ν_*.

Our analyses are slightly different for *in vitro* and *in vivo* networks, owing to the choice of η_s^X, which we consider separately below.

d. In vitro *networks*

Non-equilibrium models with absorbing states, such as the inactive state of our *in vitro* model, often fall into the directed percolation universality class [51], with exceptions when there are additional symmetries satisfied by the microscopic action [106]. The primary symmetry of the directed percolation universality class is the "rapidity symmetry." Translated into the spiking network model, rapidity symmetry would correspond to an invariance of the AEA under the transformation $\tilde{\nu}_i(t) \leftrightarrow -c\psi_i(t)$, where c is a constant chosen so that the terms $\tilde{\nu}_i(t)\psi_i(t)^2$ and $\tilde{\nu}_i(t)^2\psi_i(t)$ transform into each other, including their coefficients. The spiking network does not obey this symmetry; however, most models in the DP universality class do not exhibit rapidity symmetry exactly: it is instead an emergent symmetry that is satisfied after discarding irrelevant terms in an action tuned to the critical point [107].

To demonstrate that the spiking network model belong to the directed percolation universality class, we choose the running exponent η_s^X to impose the rapidity symmetry relationship on the couplings $g_{21,s}$ and $g_{12,s}$. Note that because all couplings $g_{mn,s}$ with $n = 0$ are initially zero, they remain so throughout the RG flow, so $g_{21,s}$ and $g_{12,s}$ are the lowest order couplings we may use to fix η_s^X. Rapidity symmetry renders $g_{21,s} = -g_{12,s}$ for all s, a hallmark of the Reggeon field theory action that describes the directed percolation universality class [51, 98]. We can then show that $\varphi_{m*}(z) = 0$, $\eta_*^X = d/4$ is a trivial fixed point that loses stability below the upper critical dimension $d_c = 4$. Below the upper critical dimension $g_{12,s}$ or $g_{21,s}$ can flow to non-zero fixed-point values $g_{12}^* = -g_{21}^*$ for a fine-tuned value of J. We assume that $\phi'(0^+) > 0$ and $\phi''(0^+) < 0$, which determines that $g_{12,s} < 0$ and hence $g_{21,s} > 0$ [108]. We will focus on $g_{21,s}$ in our presentation of the RG flow in the space of the couplings $g_{11,s}$ and $g_{21,s}$.

The running exponent η_s^X can be defined by differentiating the flow equations to derive equations for $g_{12,s}$ and $-g_{21,s}$ and equating them. This reveals that

$$\eta_s^X = \frac{d}{4} + \frac{1}{2}\frac{g_{13,s} - g_{31,s}}{1 - g_{11,s}}. \tag{C18}$$

In general, rapidity symmetry requires $g_{mn}^* = (-1)^{m+n} g_{nm}^*$ at the fixed point [98]. Because the bare action $S[\tilde{V}, V, \tilde{n}, \dot{n}]$ is not invariant under rapidity symmetry, this relation is not obeyed by $g_{mn,s}$ and $g_{nm,s}$ at finite s, but is attained in the $s \to \infty$ limit. In this limit, $\eta_*^X = d/4$ for all d and the anomalous exponent $\eta_* \propto \eta_*^X - d/4$ is always 0 within our approximation. Note that because rapidity symmetry imposes a relationship between g_{mn}^* and g_{nm}^*, to properly capture such a fixed point we must include both terms in any truncation we make; i.e., if we truncate at order z^n, we must include these terms from all nonlinearities up to $\varphi_{m=n,s}(z)$.

We can understand the qualitative features of the RG flow of the *in vitro* networks by considering only the couplings $g_{11,s}$ and $g_{21,s}$. The flow equations for these two couplings are

$$\partial_s g_{11,s} = g_{11,s} - \frac{1}{2}\frac{g_{21,s}^2}{1 - g_{11,s}} \tag{C19}$$

$$\partial_s g_{21,s} = \frac{4-d}{4} g_{21,s} - \frac{g_{21,s}^3}{(1 - g_{11,s})^2} \tag{C20}$$

The RG flow of Eqs. (C19)-(C20) in the g_{11}–g_{21} phase plane above and below the upper critical dimension $d = 4$ is shown in Fig. 8. In $d > 4$ only the trivial fixed point $(g_{11}^*, g_{21}^*) = (0, 0)$ exists, while in $d < 4$ we find the fixed point solution

$$g_{11}^* = \frac{4-d}{12-d},\; g_{21}^* = \frac{4\sqrt{4-d}}{\sqrt{d^2 - 24d + 144}},$$

with $g_{12}^* = -g_{21}^*$.

By performing a linear stability analysis around the fixed points we can estimate the correlation length exponent ν_* from the largest eigenvalue of the stability matrix, μ: $\nu_* = (2\mu)^{-1}$. The factor of 1/2 is included so that the value of ν_* matches the numerical values obtained in prior work in translation invariant systems. When $d > 4$ the trivial fixed point has one negative and one positive eigenvalue, signaling the fact we must tune only one parameter to arrive at this fixed point. The positive eigenvalue is $\mu = 1$, giving $\nu_* = 1/2$, as expected. In $d < 4$ the trivial fixed point becomes wholly unstable as it splits into the pair of non-trivial fixed points shown in Fig. 8A, which each have a stable and unstable direction, and the eigenvalue of the flow along the unstable manifold gives the correlation length exponent, which we give for $d \to 4^-$ is $\nu_* \approx 1/2 + (4-d)/16 - 7/128(4-d)^2 + \ldots$. The expansion of ν_* near $d = 4^-$ matches the one-loop perturbative estimate of ν_* for the Reggeon field theory [51].

Although within this minimal truncation we obtain an expression for ν_* valid for all $d < 4$, the expression for

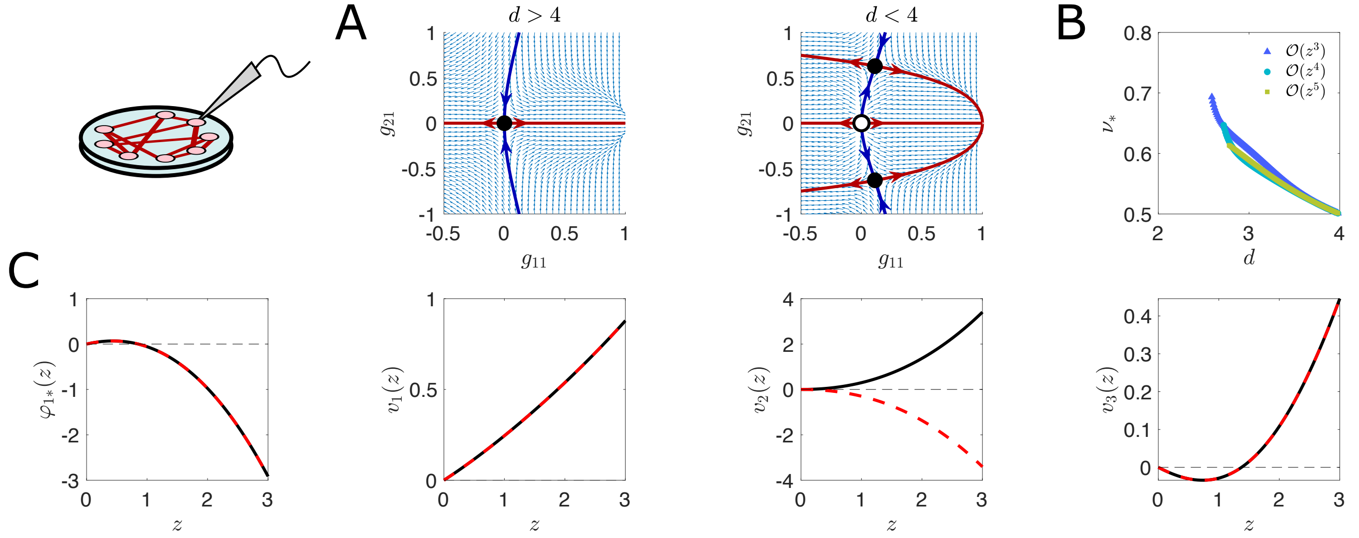

FIG. 8. **A. Renormalization group flow of the absorbing state network model** in the space of the couplings g_{11} and g_{21}, for effective dimensions $d > 4$ and $d < 4$, where 4 is the upper critical dimension. In $d > 4$ only a trivial fixed point exists, while in $d < 4$ two equivalent fixed points exist, with $g_{21}^* > 0$ selected by the initial conditions of the network model. The stable and unstable manifolds (solid lines) are colored according to the critical points (saddle nodes), with blue indicating the stable manifold and red indicating unstable manifolds. **B. Correlation length exponent for the absorbing state network** as a function of the effective dimension d. Obtained using the third (dark blue triangles), fourth (light blue circles), and fifth order (green squares) truncations. The non-perturbative analysis appears to break down at $d \lesssim 2.78$ within the local potential approximation. **C. Critical nonlinearity and eigenmodes of the directed percolation critical point** in $d = 3$, calculated up to order $m, n = 5$. Black solid lines correspond to the effective nonlinearity $\varphi_1^*(z) = \sum_{n=1}^5 \frac{g_{1n}^*}{n!} z^n$ and its eigenmodes, while red dashed curves correspond to $-\sum_{n=1}^5 \frac{g_{n,1}^*}{n!}(-z)^n$. If rapidity symmetry is obeyed, the black and red curves will match. We see that rapidity symmetry is obeyed by the critical nonlinearity and several eigenmodes, though some eigenmodes (here, $v_2(z)$) possess a different symmetry, $g_{mn}^* = (-1)^{m+n+1} g_{nm}^*$. The eigenvalues of the modes are $\mu_1 = (2\nu_*)^{-1} \approx 0.85, \mu_2 \approx 0, \mu_3 \approx -0.28$.

ν_* begins decreasing non-monotonically as d is lowered below ~ 3.2. This non-monotonic behavior is an artifact of the truncation, as we confirm by increasing the truncation up to order z^5, beyond which the calculations become computationally expensive. We indeed still find the non-trivial fixed point with rapidity symmetry, for which we estimate the critical exponent ν_* by a linear stability analysis in the higher dimension phase space. We confirm that the fixed point with rapidity symmetry is not unstable to some other fixed point lacking that symmetry: the DP fixed point has only a single relevant direction for $2.78 \lesssim d < 4$. For $d \to 2.78^+$ the estimates for ν_* diverge for our z^3 and z^4 truncations, and numerically generates erroneous complex eigenvalues at lower d in our z^5 truncation, as shown in Fig. 8B. In Sec. III we showed that the critical exponents of the directed percolation universality class in $d = 2$ successfully yield a scaling collapse, indicating that the critical phenomena are still controlled by the DP fixed point in $d < 2.78$. It is well-known in the NPRG literature that the LPA tends to break down far below the upper critical dimension, when the value of the anomalous exponent grows larger, so this may be an indication of the breakdown of the LPA for the *in vitro* spiking model.

In Fig. 8C we plot the critical nonlinearity in $d = 3$, estimated from a truncation up to $\mathcal{O}(z^5)$, along with the corresponding components of the eigenmodes of the linear stability analysis around this fixed point. We plot these against the function $-\tilde{\varphi}_{1*}(-z)$, where $\tilde{\varphi}_{1*}(z) = \sum_{m=1}^{\infty} \frac{g_{m,1}^*}{m!} z^m$. If rapidity symmetry is obeyed by the fixed point, we expect $\varphi_1^*(z) = -\tilde{\varphi}_{1*}(-z)$, and similarly for the eigenmodes. We see that one of the eigenmodes does not obey rapidity symmetry, which is to be expected because the bare action $S[\tilde{V}, V, \tilde{n}, \dot{n}]$ does not obey this symmetry. This particular eigenmode is *marginal*, having an eigenvalue numerically on the order of 10^{-14} (which appears to hold in any dimension $d < 4$), though many irrelevant eigenmodes with $\mu < 0$ also lack rapidity symmetry (not shown). Empirically, the modes lacking rapidity symmetry appear to obey a different symmetry between coefficients, $v(z) = -\tilde{v}(-z)$. While it is possible that another critical point exists possessing this symmetry, it appears to be unstable with respect to the DP fixed point, at least in dimensions $2 \leq d \leq 4$.

e. In vivo *networks*

We now consider the case of spontaneously active networks. The fact that there is a membrane potential-

independent component of the fluctuations in the spontaneous networks suggests we should choose the running exponent η_s^X to keep the coupling $g_{20,s} = 1$ for all s. This condition is tantamount to choosing the Gaussian part of the action to be invariant under the RG procedure. To enforce this restriction we find that η_s^X must be set equal to

$$\eta_s^X = \frac{d+2}{4} - \frac{\zeta_s g_{21,s}}{2} + \frac{1}{4}\frac{g_{22,s}}{1-g_{11,s}}, \tag{C21}$$

where the condition that $g_{12,s} = 0$ for all s gives that ζ_s must be equal to

$$\zeta_s = -\frac{1}{2}\frac{g_{14,s} - 2g_{13,s}g_{21,s}}{(1-g_{11,s})g_{13,s}}. \tag{C22}$$

The trivial fixed point solution is $\varphi_{m\neq 2*}(z) = 0, \varphi_{2*}(z) = 1$, $\eta_*^X = \frac{d+2}{4}$, $\zeta_* = 0$. Note that because of the factor $g_{14,s}/g_{13,s}$, we must carefully show that $g_{14,s} \to 0$ faster than $g_{13,s} \to 0$.

A linear stability analysis of this fixed point shows that the coupling $g_{11,s}$ is unstable at any d, and its initial condition must be fine-tuned (by tuning J) to bring the network to the critical point. As d is lowered below 4 the coupling $g_{13,s}$ becomes relevant, and the celebrated Wilson-Fisher (WF) fixed point emerges from the trivial fixed point. The WF fixed point controls the critical properties of the Ising model universality class, and hence *in vivo* networks are in this universality class as well. The WF fixed point has an inversion symmetry $\mathbb{Z}_2$, which translates into evenness or oddness of the effective nonlinearities, $\varphi_{m*}(-z) = (-1)^m\varphi_{m*}(z)$. If this were a symmetry of the bare action $S[\tilde{V}, V, \tilde{n}, \dot{n}]$, it would correspond to an invariance of the action under the transformation $(\tilde{n}, V-\theta) \leftrightarrow (-\tilde{n}, -(V-\theta))$. However, the action (C1) does not possess this symmetry: the size of spiking fluctuations at low firing rates are different from those at high firing rates. Because symmetry breaking terms are normally relevant, and will drive RG flows away from the critical point, we might worry that the WF fixed point cannot be seen in the spiking network model. However, our linear stability analysis of the WF fixed point confirms that the WF fixed point does not lose stability when symmetry breaking terms are present. The $\mathbb{Z}_2$ symmetry is simply an emergent symmetry that holds close to the critical point [109].

We validate our above claims by making a minimal truncation of $\varphi_{1,s}(z) = g_{11,s}z + g_{13,s}z^3/3! + g_{14,s}z^4/4!$, $\varphi_{2,s}(z) = 1$, for which $\eta_s^X = (d+2)/4$ and $\zeta_s = g_{14,s}/(1-g_{11,s})/g_{13,s}$. We include the $g_{14,s}$ term to show that the $\mathbb{Z}_2$-symmetric RG fixed point is not unstable to this mode, despite breaking the symmetry. We do not include higher order terms in $\varphi_{2,s}(z)$, or any components of $\varphi_{m\geq 3,s}(z)$, as it can be shown that these terms are irrelevant in $d > 2$ and may be neglected in this analysis.

The system of equations for the three couplings, plugging in the expressions for η_s^X and ζ_s, is

$$\partial_s g_{11,s} = g_{11,s} + \frac{1}{2}\frac{g_{13,s}}{1-g_{11,s}}, \tag{C23}$$

$$\partial_s g_{13,s} = \frac{4-d}{4}g_{13,s} + \frac{3}{2}\frac{g_{13,s}^2}{(1-g_{11,s})^2} - \frac{1}{2}\frac{g_{14,s}^2}{(1-g_{11,s})g_{13,s}} \tag{C24}$$

$$\partial_s g_{14,s} = \frac{10-3d}{4}g_{14,s} + \frac{5g_{14,s}g_{13,s}}{(1-g_{11,s})^2}. \tag{C25}$$

The non-trivial fixed point solution is

$$g_{11}^* = \frac{4-d}{10-d},$$
$$g_{13}^* = -12\frac{4-d}{(10-d)^2},$$
$$g_{14}^* = 0.$$

We plot the RG flow in the $g_{11}-g_{13}$ plane in Fig. 9A. The linear stability analysis around this fixed point yields 3 eigenvalues, of which one is positive and two are negative when $d < 4$. The eigenvector associated with the positive eigenvalue, $v_1(z)$, obeys the $\mathbb{Z}_2$ symmetry, as does the eigenmode $v_2(z)$. The other irrelevant eigenmode, however, is an even function, breaking the $\mathbb{Z}_2$ symmetry but nonetheless not affecting the leading order critical behavior of the network. We omit the exact expressions of the eigenvalues and eigenvectors, as they are rather unwieldy and offer little insight, but for higher order truncations we plot the correlation length exponent versus dimension in Fig. 9B and the eigenmodes in $d = 3$ in Fig. 9C.

For higher order truncations of the power series we find that the estimates of ν_* appear to converge as we increase the order of the truncation, but eventually become more and more difficult to solve numerically. This said, our estimates close to $d = 3$, $\nu_* = 0.7$ at $\mathcal{O}(z^{25})$ match reasonably well with the known exponent of the Ising model universality class, $\nu_* = 0.63$.

f. The effective nonlinearity near and at criticality

Finally, to connect back to the Widom scaling forms we clarify how the effective nonlinearity $\Phi(\psi)$ relates to the dimensionless nonlinearity $\varphi_1^*(z)$ and the eigenmodes $v_{1,\ell}(z)$. In doing so, we explain why the effective nonlinearity clearly depends on the bare nonlinearity $\phi(V)$, a non-universal property of the network, even when near or at a critical point.

Consider a perturbation away from the fixed point, $\varphi_{1s}(z) = \varphi_1^*(z) + \delta\varphi_s(z)$. This perturbation is determined by the decomposition of the bare nonlinearity $\phi(V)$ into

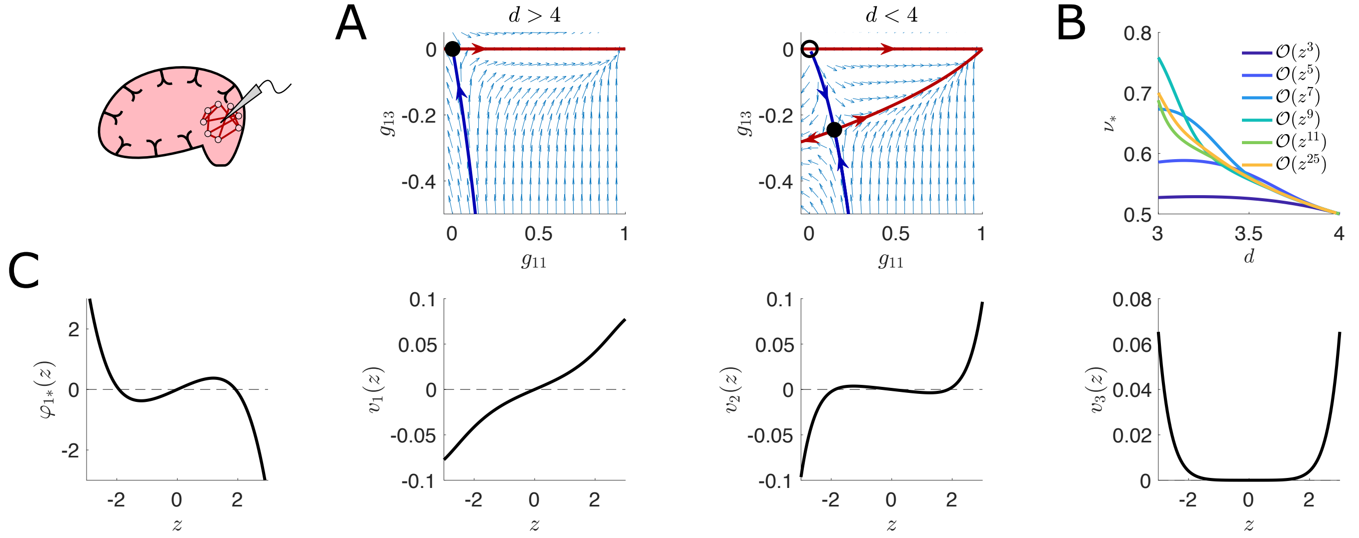

FIG. 9. **A. Renormalization group flow of the spontaneous network model** in the allowed range of the couplings g_{11} and g_{13}, for effective dimensions $d > 4$ and $d < 4$, where 4 is the upper critical dimension. In $d > 4$ only a trivial fixed point exists, while in $d < 4$ the non-trivial Wilson-Fisher fixed point enters the valid range parameter range and has exchanged stability with the trivial fixed point. The stable and unstable manifolds (solid lines) are colored according to the critical points (saddle nodes), with blue indicating the stable manifold and red indicating unstable manifolds. **B. Correlation length exponent (ν^*) estimates for the Wilson-Fisher fixed point** as a function of the effective dimension d, obtained using several truncations of the dimensionless nonlinearity $\varphi_1^*(z)$ up to order z^{25}. **C. Critical nonlinearity and eigenmodes of the Ising model critical point** in $d = 3$, calculated up to order $m = 1, n = 11$ The eigenvalues of the modes are $\mu_1 = (2\nu_*)^{-1} = 0.73, \mu_2 = -0.18, \mu_3 = -0.81$. At the Ising model fixed point the critical nonlinearity is an odd function $\varphi_1^*(-z) = -\varphi_1^*(z)$. This symmetry is also obeyed by several eigenmodes, while other eigenmodes are even. Despite breaking the $\mathbb{Z}_2$ symmetry, the even eigenmodes are irrelevant at the fixed point.

a series of eigenmodes near the fixed point:

$$\varphi_{1,s}(z) = \varphi_1^*(z) + \sum_\ell c_\ell e^{\mu_\ell s} v_{1,\ell}(z), \tag{C26}$$

where μ_ℓ are the eigenvalues, which are non-integer in general, $v_{1,\ell}(z)$ is the corresponding eigenmode to the $\varphi_{1,s}(z)$ component, and c_ℓ are the loadings onto each of these eigenmodes. In order for the model to be at a critical point the loadings c_ℓ corresponding to the relevant directions with positive eigenvalues $\mu_\ell > 0$ must be tuned to zero in order for the RG dynamics to lie on the critical manifold. The irrelevant directions, corresponding to negative eigenvalues $\mu_\ell < 0$, will decay as $s \to \infty$. At the critical points we focus on in this work there are two modes with positive eigenvalues. The first is the trivial constant mode $v_{1,0}(z) \propto 1$, which only contributes to the running baseline rate Π_s and will not drive the dimensionless nonlinearity further away from the critical manifold. The loading c_1 of the other mode with positive eigenvalue determines how close the network is tuned to the critical point, and the "correlation length exponent" ν_* is derived from this eigenvalue by the definition $\mu_1 \equiv (2\nu_*)^{-1}$. The flow of the nonlinearity only depends on the synaptic weight J (through the maximum eigenvalue Λ_{max}), not $\mathcal{E}$, so we expect $c_1 \propto J_c - J$ close to the critical point.

The effective nonlinearity $\Phi(\psi)$ is related to the dimensionless nonlinearity by

$$\begin{aligned}\Phi(\psi) &- \nu_c - \Lambda_{\text{max}}^{-1}(\psi - \psi_c) \\ &= \lim_{s\to\infty} e^{-s(d/2+1-\eta_*^X)} \varphi_{1,s}\left((\psi - \psi_c)e^{s(d/2-\eta_*^X)}\right).\end{aligned}$$

In *in vitro* networks both $\Pi_s \to \nu_c$ and $\theta_s \to \psi_c$ may be set to 0. To see how the RG critical point shapes the effective nonlinearity, we first plug in the expansion (C26).

$$\begin{aligned}&\Phi(\psi) - \nu_c - \Lambda_{\text{max}}^{-1}(\psi - \psi_c) \\ &\sim e^{-s(d/2+1-\eta_*^X)} \varphi_{1s}\left((\psi - \psi_c)e^{s(d/2-\eta_*^X)}\right) \\ &= e^{-s(d/2+1-\eta_*^X)} \\ &\quad \times \Bigg\{ c_1 e^{\frac{s}{2\nu_*}} v_1\left((\psi - \psi_c)e^{s(d/2-\eta_*^X)}\right) \\ &\qquad + \varphi_1^*\left((\psi - \psi_c)e^{s(d/2-\eta_*^X)}\right) \\ &\qquad + \sum_{\ell\geq 2} c_\ell e^{\mu_\ell s} v_{1,\ell}\left((\psi - \psi_c)e^{s(d/2-\eta_*^X)}\right)\Bigg\},\end{aligned}$$

where we have separated out the $\ell = 1$ term from the eigenmodes. We can understand the origin of scaling by imagining that for $c_1 \propto J_c - J \neq 0$ we only run the RG flow out to an RG-time s such that $|J_c - J|e^{\frac{s}{2\nu_*}} = \text{const}$.

We can thus replace $e^s \propto |J_c - J|^{-2\nu_*}$ in the eigenmode expansion above, which yields

$$\Phi(\psi) - \nu_c - \Lambda_{\max}^{-1}(\psi - \psi_c) \sim |J_c - J|^{2\nu_*(d/2+1-\eta_*^X)} f^* \left((\psi - \psi_c)|J_c - J|^{-2\nu_*(d/2-\eta_*^X)}\right) + \sum_{\ell \geq 2} c_\ell |J_c - J|^{2\nu_*(d/2+1-\eta_*^X-\mu_\ell)} v_{1,\ell}\left((\psi - \psi_c)|J_c - J|^{-2\nu_*(d/2-\eta_*^X)}\right), \quad \text{(C27)}$$

where $f^*(z) \equiv \text{const.} \times v_1(z) + \varphi_*(z)$. To take stock of what we have derived, Eq. (C27) relates the effective nonlinearity $\Phi(\psi)$—which depends on the details of the bare nonlinearity $\phi(V)$—to the detail-independent universal properties of the RG fixed point: the fixed point function $\varphi_*(z)$, eigenmodes $v_\ell(z)$, and the critical exponents ν_*, η_*^X, and μ_ℓ. These universal quantities depend only on the properties of the RG critical point. The non-universal terms on the right-hand-side of Eq. (C27) are the loadings c_ℓ, which describe the initial projection of the bare nonlinearity $\phi(V)$ on to the eigenmodes of the RG critical point [110]. Thus, we see that non-universal information about the microscopic features of the model enter through the loadings c_ℓ associated with the corrections to scaling, and ultimately shape the effective nonlinearity $\Phi(\psi)$. Importantly, although the RG critical point possesses an emergent $\mathbb{Z}_2$ symmetry, this symmetry is only inherited by $\varphi_1^*(z)$, not the eigenmodes $v_{1,\ell}(z)$, due to the fact that the bare action is not $\mathbb{Z}_2$ symmetric. This is why the nonlinearities $\Phi(\psi) - \nu_c$ we observe in our simulations and by solving Eq. (C11) are not odd functions in $\psi - \psi_c$, even though the bare nonlinearity $\phi(V) - \phi(\theta)$ is an odd function in $V - \theta$.

As the network is tuned to its critical point at $J = J_c$, the critical nonlinearity retains its dependence on the non-universal c_ℓ and its lack of an overall $\mathbb{Z}_2$ symmetry. One can show the asymptotic behaviors $\varphi_*(z) \sim \mathcal{A}_d z^{1+\frac{1}{d/2-\eta_*^X}}$ and $v_{1,\ell}(z) \sim \mathcal{B}_\ell z^{1+\frac{1-\mu_\ell}{d/2-\eta_*^X}}$ as $|z| \to \infty$, and the factors of $J_c - J$ ultimately cancel out of Eq. (C27) to yield the critical nonlinearity

$$\Phi(\psi) - \nu_c - \Lambda_{\max}^{-1}(\psi - \psi_c) \sim \mathcal{A}_d(\psi - \psi_c)^{1+\frac{1}{d/2-\eta_*^X}} + \sum_{\ell \geq 2} c_\ell \mathcal{B}_\ell (\psi - \psi_c)^{1+\frac{1+|\mu_\ell|}{d/2-\eta_*^X}}, \quad \text{(C28)}$$

which still depends on the non-universal loadings c_ℓ, but is now a series in *non-analytic* powers of $\psi - \psi_c$. For $\psi - \psi_c < 0$ these powers should be interpreted as the power of the absolute value of $|\psi - \psi_c|$, with an overall sign that depends on whether $v_{1,\ell}(z)$ is odd or even.

Plugging Eq. (C27) into the dynamical equation (12) for the mean membrane potential $\psi(t)$ and keeping only the leading scaling function $f^*(z)$ (the subleading terms contribute *corrections to scaling* [18, 111]), we obtain an implicit solution for $\psi(t) - \psi_c$ that can be formally inverted to give the Widom scaling forms (Eqs. (4)-(6)) with $\beta_* = \frac{\nu_* d}{2}$ and $\Delta_* = \frac{\nu_*}{2}(d+4)$ in *in vitro* networks and $\beta_* = \frac{\nu_*}{2}(d-2)$ and $\Delta_* = \frac{\nu_*}{2}(d+2)$ in *in vivo* networks. Comparing these expressions with the general scaling relations expected in the directed percolation ($\beta_* = \frac{\nu_*}{2}(d+\eta_*)$, $\Delta_* = \frac{\nu_*}{2}(d+2z_*-\eta_*)$) [51] and Ising ($\beta_* = \frac{\nu_*}{2}(d-2+\eta_*)$ and $\Delta_* = \frac{\nu_*}{2}(d+2-\eta_*)$) universality classes, we confirm that our LPA approximation predicts trivial (mean-field) values of $z_* = 2$ and $\eta_* = 0$ for the dynamical and anomalous exponents [112].

Because we identified the critical points we have found within the LPA with the well-known universality classes of directed percolation and the Ising model, in performing our scaling collapses in Sec. III we were able to use the known values of the critical exponents, including the non-trivial values of the anomalous exponent η_* and dynamical exponent z_*, for the $2d$ and $3d$ lattices. In our excitatory-inhibitory networks with excitatory random regular connections we used our LPA estimates as starting points for determining the values of the critical exponents that collapsed our data. This demonstrates that even when networks lack translation invariance this may not drastically change the critical exponents. That said, higher degree random regular networks with global inhibition proved to have mean-field scaling, suggesting that proper treatment of effects beyond the LPA are necessary to determine whether these networks can again be tuned to have anomalous scaling.
